# Supplementary material for: Benzoxazine–Purine Hybrids as Antiproliferative Agents: Rational Design and Divergent Mechanisms of Action
Source: Pharmaceutics. 2025 Sep 26;17(10):1260. doi: 10.3390/pharmaceutics17101260 (PMC12567375; doi:10.3390/pharmaceutics17101260)
Supplement: Supplementary file 1 [file pharmaceutics-17-01260-s001.zip › pharmaceutics-3883064-supplementary.pdf]

Supplementary Materials

Benzoxazine–Purine Hybrids as Antiproliferative Agents: Rational Design and Divergent Mechanisms of Action

Houria Boulaiz [1,2,3‡], Yaiza Jiménez-Martínez [2,3‡], Francisco Franco-Montalbán [4], Jesús Peña-Martín [2,3], Ana Conejo-García [2,4,*] and M. Dora Carrión [2,4,*]

[1] Department of Human Anatomy and Embryology, Faculty of Medicine, University of Granada, 18016 Granada, Spain; hboulaiz@ugr.es
2 Biosanitary Institute of Granada (ibs.GRANADA), SAS-University of Granada, Avenida de Madrid, 15, 18012, Granada, Spain; yaijmartinez@correo.ugr.es (Y.J.-M.); jespmar@ugr.es (J.P.M.);
3 Biopathology and Regenerative Medicine Institute (IBIMER), Centre for Biomedical Research, University of Granada, 18016 Granada, Spain;
4 Department of Medicinal and Organic Chemistry and Excellence Research Unit of Chemistry Applied to Biomedicine and the Environment, Faculty of Pharmacy, Campus Cartuja s/n, 18071, University of Granada, Granada, Spain; ffranco@ugr.es
‡ These authors contributed equally.
* Correspondence: authors: aconejo@ugr.es (A.C.-G.); dcarrion@ugr.es (M.D.C.)

Table of contents

1. Structural Determination of Synthesized Compounds 2

1.1. Characterization by ^{1}H NMR, ^{13}C NMR and HRMS 2

1.2. NMR Spectra 8

1.3. HRMS Spectra 26

2. Biology 35

2.1. IC_{50} Curves for Compounds **3–14** 35

2.2. Kinase inhibition assay 36

2.3. Comparative analysis of the binding between compounds **2** and 1**2** 39

3. References 42

1. Structural Determination of Synthesized Compounds

1.1. Characterization by ^{1}H NMR, ^{13}C NMR and HRMS

(6 or 7 Substituted-3,4-dihydro-2*H*-benzo[b][1,4]oxazin-2-yl)methanol (compounds 18-20)

(7-Chloro-3,4-dihydro-2*H*-benzo[b][1,4]oxazin-2-yl)methanol (**18**). Brown oil; 74% yield. ^{1}H NMR (500 MHz, $CDCl_3$): δ (ppm): 6.81 (d,1H,*J* = 2.3 Hz, H_{benz}), 6.73 (dd, 1H, *J* = 8.4, 2.3 Hz, H_{benz}), 6.51 (d, 1H, *J* = 8.4 Hz, H_{benz}), 4.23 - 4.19 (m, 1H, $H_{oxazine}$), 3.86 - 3.77 (m, 2H, C$\underline{H_2}$OH), 3.38 - 3.28 (m, 2H, $H_{oxazine}$). ^{13}C NMR (126 MHz, $CDCl_3$): δ (ppm): 144.0 (C_{benz}), 132.0 (C_{benz}), 123.3 (C_{benz}), 121.4 (CH_{benz}), 117.1 (CH_{benz}), 116.1 (CH_{benz}), 74.6 ($CH_{oxazine}$), 63.3 (CH_2OH), 41.9 ($CH_{2oxazine}$). HRMS (ESI) (m/z) calcd for $C_9H_{11}ClNO_2$ $[M+H]^+$ 200.0478; found 200.0490.

(6-Bromo-3,4-dihydro-2*H*-benzo[b][1,4]oxazin-2-yl)methanol (**19**) [1]. Brown oil; 75% yield. ^{1}H NMR (500 MHz, $CDCl_3$) δ (ppm): 6.75 - 6.72 (m, 2H, H_{benz}), 6.67 (d, 1H, *J* = 8.4 Hz, H_{benz}), 4.21 - 4.18 (m, 1H, $H_{oxazine}$), 3.86 - 3.78 (m, 2H, C$\underline{H_2}$OH), 3.38 – 3.28 (m, 2H, $H_{oxazine}$). ^{13}C NMR (126 MHz, $CDCl_3$) δ (ppm) 142.4 (C_{benz}), 134.5 (C_{benz}), 121.3 (CH_{benz}), 120.6 (C_{benz}), 118.1 (CH_{benz}), 117.6 (CH_{benz}), 74.2 ($CH_{oxazine}$), 63.2 (CH_2OH), 41.6 ($CH_{2oxazine}$). HRMS (ESI) (m/z) calcd for $C_9H_{11}NO_2Br$ $[M+H]^+$ 243.9973; found 243.9993.

(6-Methyl-3,4-dihydro-2*H*-benzo[b][1,4]oxazin-2-yl)methanol (**20**) [2]. Brown oil; 84%.^{1}H NMR (400 MHz, $CDCl_3$) δ (ppm) 6.70 (d, 1H, *J* = 8.1 Hz, H_{benz}), 6.48 - 6.45 (m, 1H, H_{benz}), 6.41 (d, 1H, *J* = 2.0 Hz, H_{benz}), 4.20 - 4.17 (m, 1H, $H_{oxazine}$), 3.84 - 3.75 (m, 2H, C$\underline{H_2}$OH), 3.35 - 3.24 (m, 2H, $H_{oxazine}$), 2.20 (s, 3H, CH_3). ^{13}C NMR (101 MHz, $CDCl_3$) δ (ppm) 141.3 (C_{benz}), 132.7 (C_{benz}), 130.8 (C_{benz}), 119.6 (CH_{benz}), 116.4 (CH_{benz}), 116.0 (CH_{benz}), 74.3 ($CH_{oxazine}$), 63.4 (CH_2OH), 42.1 ($CH_{2oxazine}$), 20.7 (CH_3). HRMS (ESI) (m/z) calcd for $C_{10}H_{14}NO_2$ $[M+H]^+$ 180.1025; found 180.1010.

(6 or 7 Substituted-4-tosyl-3,4-dihydro-2*H*-benzo[b][1,4-]oxazin-2-yl)methanol (compounds 21-23)

(7-Chloro-4-tosyl-3,4-dihydro-2*H*-benzo[b][1,4-]oxazin-2-yl)metanol (**21**). Brown oil; 87% yield. ^{1}H NMR (500 MHz, $CDCl_3$) δ (ppm) 7.75 (d,1H, *J* = 8.9 Hz, H_{benz}), 7.52 (d, 2H, *J* = 8.3 Hz, 2 × H_{tosyl}), 7.25 (d, 2H, *J* = 8.3 Hz, 2 × H_{tosyl}), 6.91 (dd,1H,*J* = 8.9, 2.4 Hz, H_{benz}), 6.84 (d,1H, *J* = 2.4 Hz, H_{benz}), 4.26 (dd,1H, *J* = 14.5, 2.4 Hz, $H_{oxazine}$), 3.75 – 3.63 (m, 2H, C$\underline{H_2}$OH), 3.48 – 3.44 (m, 1H, $H_{oxazine}$), 3.33 (dd, 1H, *J* = 14.5, 10.1 Hz,1H, $H_{oxazine}$), 2.39 (s, 3H, CH_{3tosyl}). ^{13}C NMR (126 MHz, $CDCl_3$) δ (ppm) 147.4 (C_{tosyl}), 144.7 (C_{benz}), 135.4 (C_{tosyl}), 131.3 (C_{benz}), 130.2 (2 × CH_{tosyl}), 127.3 (2

× CH_{tosyl}), 125.5 (CH_{benz}), 122.4 (C_{benz}), 121.4 (CH_{benz}), 117.6 (CH_{benz}), 72.3 (CHoxazine), 62.5 (CH_2OH), 45.4 ($CH_{2oxazine}$), 21.8 (CH_{3tosyl}). HRMS (ESI) (m/z) calcd for $C_{16}H_{16}NO_4NaSCl$ $[M+Na]^+$ 376.0386, found 376,0426.

(6-Bromo-4-tosyl-3,4-dihydro-2*H*-benzo[b][1,4-]oxazin-2-yl)methanol (**22**). Brown oil; 89% yield. ^1H NMR (500 MHz, $CDCl_3$) δ (ppm) 8.00 (d, 1H, *J* = 2.3 Hz, 1H, H_{benz}), 7.64 – 7.52 (m, 2H, 2 × H_{tosyl}), 7.34 – 7.24 (m, 2H, 2 × H_{tosyl}), 7.17 (dd, 1H, *J* = 8.7, 2.3 Hz, H_{benz}), 6.73 (d,1H, *J* = 8.7 Hz, H_{benz}), 4.26 (dd, 1H, *J* = 14.4, 2.4 Hz, $H_{oxazine}$), 3.78 - 3.69 (m, 2H, C$\underline{H}_2$OH), 3.52 – 3.49 (m, 1H, $H_{oxazine}$), 3.34 (dd, 1H, *J* = 14.4, 9.9 Hz, $H_{oxazine}$), 2,42 (s, 3H, CH_{3tosyl}). ^{13}C NMR (126 MHz, $CDCl_3$) δ (ppm) 145.8 (C_{tosyl}), 144.7 (C_{benz}), 135.3 (C_{tosyl}), 130.1 (2 × CH_{tosyl}), 129.0 (CH_{benz}), 127.2 (2 × CH_{tosyl}), 126.7 (CH_{benz}), 124.9 (C_{benz}), 118.8 (CH_{benz}), 112.9 (C_{benz}), 72.2 ($CH_{oxazine}$), 62.5 (CH_2OH), 45.2 ($CH_{2oxazine}$), 21.6 (CH_{3tosyl}). HRMS (ESI) (m/z) calcd for $C_{16}H_{16}BrNO_4SCl$ $[M+Cl]^-$ 431.9672; found 431.9668.

(6-Methyl-4-tosyl-3,4-dihydro-2*H*-benzo[b][1,4-]oxazin-2-yl)methanol (**23**). Brown oil; 89% yield. ^1H NMR (500 MHz, $CDCl_3$) δ (ppm) 7.64 (d,1H,*J* = 1.6 Hz, H_{benz}), 7.54 (d, 2H, *J* = 8.3 Hz, 2 × H_{tosyl}), 7.24 (d, 2H, *J* = 8.3 Hz, 2 × H_{tosyl}), 6.88 (dd,1H, *J* = 8.5, 2.0 Hz, H_{benz}), 6.73 (d, 1H, *J* = 8.3 Hz, H_{benz}), 4.25 (dd,1H,*J* = 14.4, 2.5 Hz, $H_{oxazine}$), 3.74 - 3.62 (m, 2H, CH_2OH), 3.56 – 3.46 (m, 1H, $H_{oxazine}$), 3.34 (dd, 1H, *J* = 14.4, 10.0 Hz, $H_{oxazine}$), 2.39 (s, 3H, CH_{3tosyl}), 2.32 (s, 3H, CH_3). ^{13}C NMR (126 MHz, $CDCl_3$) δ (ppm) 144.6 (C_{benz}), 144.3 (C_{benz}), 135.7 (C_{tosyl}), 129.9 (2 × CH_{tosyl}), 127.2 (2 × CH_{tosyl}), 127.2 (C_{benz}), 127.0 (CH_{benz}), 124.6 (CH_{benz}), 117.00 (CH_{benz}), 71.8 ($CH_{oxazine}$), 62.6 (CH_2OH), 45.6 ($CH_{2oxazine}$), 21.6 (CH_{3tosyl}), 20.8 (CH_3). HRMS (ESI) (m/z) calcd for $C_{17}H_{19}NO_4NaS$ $[M+Na]^+$ 356.0932; found 356.0915.

Substituted 2-((6-halo or 2,6-dihalo-9*H*-purin-9-yl)methyl)-4-tosyl-3,4-dihydro-2*H*-benzo[b][1,4]oxazine derivatives (compounds 3-6, 7-9, 11-13)

7-Chloro-2-((6-chloro-9*H*-purin-9-yl)methyl)-4-tosyl-3,4-dihydro-2*H*-benzo[b][1,4]oxazine (**3**). Brown solid; 80% yield; mp: 159 - 160ºC. ^1H NMR (400 MHz, $CDCl_3$) δ (ppm) 8.77 (s, 1H, H_{purine}), 8.11 (s, 1H, H_{purine}), 7.71 (d,1H,*J* = 8.9 Hz, H_{benz}), 7.48 (d, 2H, *J* = 8.1 Hz, 2 × H_{tosyl}), 7.21 (d, 2H, *J* = 8.1 Hz, , 2 × H_{tosyl}), 6.94 (dd,1H,*J* = 8.9, 2.4 Hz, H_{benz}), 6.83 (d, 1H, *J* = 2.4 Hz, H_{benz}), 4.51 (dd, 1H, *J* = 14.5, 3.6 Hz, $H_{oxazine}$), 4.40 – 4.34 (m, 2H, -CH_2-), 3.78 – 3.74 (m, 1H, $H_{oxazine}$), 3.08 (dd,1H, *J* = 14.5, 9.8 Hz, Hoxazine), 2.35 (s, 3H, CH_{3tosyl}). ^{13}C NMR **(**101 MHz, $CDCl_3$) δ (ppm) 152.3 (CH_{purine}), 151.9 (C_{purine}), 151.6 (C_{purine}), 146.2 (C_{benz}), 145.7 (CH_{purine}), 145.0 (C_{tosyl}), 135.1

(C_{tosyl}), 131.8 (C_{benz}), 131.6 (C_{purine}), 130.4 (2 × CH_{tosyl}), 127.3 (2 × CH_{tosyl}), 125.6 (CH_{benz}), 122.3 (CH_{benz}), 122.2 (C_{benz}), 117.7 (CH_{benz}), 69.9 ($CH_{oxazine}$), 45.8 ($CH_{2oxazine}$), 45.4 (-CH_2-), 21.7 (CH_3). HRMS (ESI) (m/z) calcd for $C_{21}H_{18}N_5O_3SCl_2$ $[M+H]^+$ 490.0507; found 490.0499.

7-Chloro-2-((2,6-dichloro-9*H*-purin-9-yl)methyl)-4-tosyl-3,4-dihydro-2*H*-benzo[b][1,4]oxazine (**4**). White solid; 83% yield; mp: 90 - 92ºC. ^1H NMR (500 MHz, $CDCl_3$) δ (ppm) 8.11 (s, 1H, H_{purine}), 7.73 (d,1H,*J* = 8.9 Hz, H_{benz}), 7.51 (d, 2H, *J* = 8.3 Hz, 2 × H_{tosyl}), 7.37 – 7.16 (m, 2H, 2 × H_{tosyl}), 6.95 (dd,1H,*J* = 8.9, 2.4 Hz, H_{benz}), 6.81 (d, 1H, *J* = 2.4 Hz, H_{benz}), 4.50 (d, 1H, *J* = 3.3 Hz, $H_{oxazine}$), 4.40 – 4.30 (m, 2H, -CH_2-), 3.75 – 3.70 (m,1H, $H_{oxazine}$), 3.18 – 3.15 (m, 1H, $H_{oxazine}$), 2.36 (s, 2H, CH_{3tosyl}). ^{13}C NMR (126 MHz, $CDCl_3$) δ (ppm) 153.2 (C_{purine}), 153.0 (C_{purine}), 152.2 (C_{purine}), 146.3 (CH_{purine}), 145.9 (C_{benz}), 145.0 (C_{tosyl}), 134.7 (C_{tosyl}), 131.6 (C_{benz}), 130.6 (C_{purine}), 130.3 (2 × CH_{tosyl}), 127.1 (2 × CH_{tosyl}), 125.4 (CH_{benz}), 122.2 (CH_{benz}), 122.0 (C_{benz}), 117.5 (CH_{benz}), 69.4 ($CH_{oxazine}$), 45.53($CH_{2oxazine}$), 45.4 (-CH_2-), 21.6 (CH_{3tosyl}). HRMS (ESI) (m/z) calcd for $C_{21}H_{17}N_5O_3SCl_3$ $[M+H]^+$ 524.0118; found 524.0153.

7-Chloro-2-((6-bromo-9*H*-purin-9-yl)methyl)-4-tosyl-3,4-dihydro-2*H*-benzo[b][1,4]oxazine (**5**). Brown oil; 85% yield; mp: 170 - 172ºC. ^1H NMR (400 MHz, $CDCl_3$) δ (ppm) 8.69 (s, 1H, H_{purine}), 8.11 (s, 1H, H_{purine}), 7.68 (d, 1H, *J* = 8.9 Hz, H_{benz}), 7.45 (d, 2H, *J* = 8.3 Hz, 2 × H_{tosyl}), 7.19 (d, 2H, *J* = 8.3 Hz, 2 × H_{tosyl}), 6.91 (dd,1H, *J* = 8.9, 2.4 Hz, H_{benz}), 6.79 (d,1H, *J* = 2.4 Hz, H_{benz}), 4.48 (dd,1H, *J* = 14.5, 3.6 Hz, $H_{oxazine}$), 4.37 – 4.32 (m, 2H, -CH_2-), 3.74 – 3.72 (m,1H, $H_{oxazine}$), 3.06 (dd,1H, *J* = 14.5, 9.8 Hz, $H_{oxazine}$) 2.33 (s, 3H, -CH_{3tosyl}). ^{13}C NMR (101 MHz, $CDCl_3$) δ (ppm) 152.0, (CH_{purine}), 150.5 (C_{purine}), 146.0 (C_{benz}), 145.4 (CH_{purine}), 144.8 (C_{tosyl}), 143.5 (C_{purine}), 134.9 (C_{tosyl}), 134.0 (C_{purine}), 131.6 (C_{benz}), 130.2 (2 × CH_{tosyl}), 127.1 (2 × CH_{tosyl}), 125.4 (CH_{benz}), 122.0 (CH_{benz}), 122.0 (C_{benz}), 117.5 (CH_{benz}), 69.7 ($CH_{oxazine}$), 45.6 ($CH_{2oxazine}$), 45.2 (-CH_2-), 21.6 (CH_{3tosyl}). HRMS (ESI) (m/z) calcd for $C_{21}H_{18}N_5O_3SClBr$ $[M+H]^+$ 534.0002; found 533.9993.

6-Bromo-2-((6-chloro-9*H*-purin-9-yl)methyl)-4-tosyl-3,4-dihydro-2*H*-benzo[b][1,4]oxazine (**7**). White solid; 83% yield; mp: 180 - 181ºC. ^1H NMR (400 MHz, $CDCl_3$) δ (ppm) 8.77 (s, 1H, H_{purine}), 8.16 (s, 1H, H_{purine}), 7.93 (d,1H,*J* = 2.3 Hz, H_{benz}), 7.63 – 7.37 (m, 2 × H_{tosyl}), 7.23 (d, 2H, *J* = 8.1 Hz, 2 × H_{tosyl}), 7.17 (dd,1H, *J* = 8.8, 2.3 Hz, H_{benz}), 6.69 (d,1H,*J* = 8.8 Hz, H_{benz}), 4.55 (dd, 1H, *J* = 14.5, 3.5 Hz, $H_{oxazine}$), 4.43 – 4.34 (m, 2H, -CH_2-), 3.81 - 3.77 (m, 1H, $H_{oxazine}$), 3.09 (d,1H,*J* = 14.5, 9.5 Hz, $H_{oxazine}$), 2.36 (s, 3H, CH_{3tosyl}). ^{13}C NMR (101 MHz, $CDCl_3$) δ (ppm) 152.1 (CH_{purine}), 151.7 (C_{purine}), 151.4 (C_{purine}), 145.7 (CH_{purine}), 145.0 (C_{tosyl}), 144.6 (C_{benz}), 134.7 (C_{tosyl}), 131.3 (C_{purine}), 130.2 (2 × CH_{tosyl}), 129.3 (CH_{benz}), 127.1 (2 × CH_{tosyl}), 126.7 (CH_{benz}), 124.5 (C_{benz}), 118.9 (CH_{benz}), 113.7

(C_{benz}), 69.7 ($CH_{oxazine}$), 45.5 ($CH_{2oxazine}$), 45.2 (-CH_2-), 21.6 (-CH_{3tosyl}). HRMS (ESI) (m/z) calcd for $C_{21}H_{18}N_5O_3SClBr$ $[M+H]^+$ 534.0002; found 533.9969.

6-Bromo-2-((2,6-dichloro-9*H*-purin-9-yl)methyl)-4-tosyl-3,4-dihydro-2*H*-benzo[b][1,4]oxazine (**8**). White solid; 85% yield; mp: 108 - 110ºC. ^1H NMR (400 MHz, $CDCl_3$) δ (ppm) 8.12 (s, 1H, H_{purine}), 7.98 (d, 1H, *J* = 2.3 Hz, H_{benz}), 7.57 (d,2H, *J* = 8.1 Hz, 2 × H_{tosyl}), 7.30 (d, 2H, *J* = 8.6 Hz, 2 × H_{tosyl}), 7.19 (dd, 1H, *J* = 8.7, 2.3 Hz, H_{benz}), 6.69 (d, 1H, *J* = 8.7 Hz, H_{benz}), 4.53 (dd, 1H, *J* = 14.4, 3.3 Hz, $H_{oxazine}$), 4.40 – 4.28 (m, 2H, -CH_2-), 3.79 - 3.75 (m, 1H, $H_{oxazine}$), 3.19 (d, 1H, *J* = 14.4, 9.5 Hz, $H_{oxazine}$), 2.38 (s, 3H, CH_{3tosyl}). ^{13}C NMR (101 MHz, $CDCl_3$) δ (ppm) 153.3 (C_{purine}), 153.0 (C_{purine}), 152.2 (C_{purine}), 146.3 (CH_{purine}), 145.1 (C_{tosyl}), 144.4 (C_{benz}), 134.7 (C_{tosyl}), 130.6 (C_{purine}), 130.3 (2 × CH_{tosyl}), 129.4 (CH_{benz}), 127.2 (2 × CH_{tosyl}), 126.8 (CH_{benz}), 124.6 (C_{benz}), 118.8 (CH_{benz}), 113.9 (C_{benz}), 69.5 ($CH_{2oxazine}$), 45.5 ($CH_{2oxazine}$, -CH_2-), 21.6 (CH_{3tosyl}). HRMS (ESI) (m/z) calcd for $C_{21}H_{17}N_5O_3SCl_2Br$ $[M+H]^+$ 567.9613; found 567.9615.

6-Bromo-2-((6-bromo-9*H*-purin-9-yl)methyl)-4-tosyl-3,4-dihydro-2*H*-benzo[b][1,4]oxazine (**9**). White solid; 86% yield; mp: 187 – 188ºC. ^1H NMR (400 MHz, $CDCl_3$) δ (ppm) 8.74 (s, 1H, H_{purine}), 8.16 (s, 1H, Hpurine), 7.95 (d, 1H, *J* = 2.3 Hz, H_{benz}), 7.54 (d, 2H, *J* = 8.4 Hz, 2 × H_{tosyl}), 7.27 – 7.21 (m, 2H, 2 × H_{tosyl}), 7.18 (dd, 1H, *J* = 8.8, 2.4 Hz, H_{benz}), 6.70 (d, 1H, *J* = 8.8 Hz, H_{benz}), 4.54 (dd, 1H, *J* = 14.7, 3.6 Hz, $H_{oxazine}$), 4.43 - 4.34 (m, 2H, -CH_2-), 3.82 - 3.76 (m, 1H, $H_{oxazine}$), 3.15 (dd, 1H, *J* = 14.7, 9.6 Hz, $H_{oxazine}$), 2.38 (s, 3H, CH_{3tosyl}). ^{13}C NMR (101 MHz, $CDCl_3$) δ (ppm) 152.1 (CH_{purine}), 150.5 (C_{purine}), 145.5 (CH_{purine}), 145.0 (C_{tosyl}), 144.6 (C_{benz}), 143.5 (C_{purine}), 134.8 (C_{tosyl}), 134.0 (Cpurine), 130.3 (2 × CH_{tosyl}), 129.4 (CH_{benz}), 127.2 (2 × CH_{tosyl}), 126.8 (CH_{benz}), 124.5 (C_{benz}), 118.9 (CH_{benz}), 113.8 (C_{benz}), 69.8 ($CH_{oxazine}$), 45.6 ($CH_{2oxazine}$), 45.3 (-CH_2-), 21.6 (CH_{3tsoyl}). HRMS (ESI) (m/z) calcd for $C_{21}H_{18}N_5O_3SBr_2$ $[M+H]^+$ 577.9497; found 577.9446.

2-((6-Chloro-9*H*-purin-9-yl)methyl)-6-methyl-4-tosyl-3,4-dihydro-2*H*-benzo[b][1,4]oxazine (**11**). White solid; 83% yield; mp: 169 – 170ºC. ^1H NMR (400 MHz, $CDCl_3$) δ (ppm) 8.76 (s, 1H, H_{purine}), 8.16 (s, 1H, H_{purine}), 7.56 (d, 1H, *J* = 1.9 Hz, H_{benz}), 7.50 - 7.48 (m, 2 × H_{tosyl}), 7.20 (d, 2H, *J* = 8.0 Hz, 2 × H_{tosyl}), 6.86 (d,1H,*J* = 2.0 Hz, H_{benz}), 6.69 (d,1H, *J* = 8.3 Hz, H_{benz}), 4.51 (dd,1H, *J* = 14,7, 3.6 Hz, $H_{oxazine}$), 4.40 - 4.32 (m, 2H, -CH_2-), 3.78 - 3.73 (m, 1H, $H_{oxazine}$), 3.11 (dd, 1H, *J* = 14.7, 9.7 Hz, $H_{oxazine}$), 2.34 (s, 3H, CH_{3tosyl}), 2.30 (S, 3H, CH_3). ^{13}C NMR (101 MHz, $CDCl_3$) δ (ppm) 152.0 (CH_{purine}), 151.2 (C_{purine}), 151.0 (C_{purine}), 145.9 (CH_{purine}), 144.5 (C_{tosyl}), 143.4 (C_{benz}), 135.2 (C_{tosyl}), 131.3 (C_{purine}), 130.0 (C_{benz}), 130.0 (2 × CH_{tosyl}), 127.3 (CH_{benz}), 127.1 (2 × CH_{tosyl}), 124.6

(CH_{benz}), 122.8 (C_{benz}), 117.0 (CH_{benz}), 69.3 ($CH_{oxazine}$), 45.9 ($-CH_2$), 45.3 ($CH_{2oxazine}$), 21.6 (CH_{3tosyl}), 20.9 (CH_3). HRMS (ESI) (m/z) calcd for $C_{22}H_{21}N_5O_3SCl$ $[M+H]^+$ 470.1054; found 470.1042.

2-((2,6-Dichloro-9*H*-purin-9-yl)methyl)-6-methyl-4-tosyl-3,4-dihydro-2*H*-benzo[b][1,4]oxazine (**12**). White solid; 84% yield; mp: 110 – 112ºC. ^1H NMR (400 MHz, $CDCl_3$) δ (ppm) 8.15 (s, 1H, H_{purine}), 7.57 - 7.56 (m, 1H, H_{purine}), 7.51 - 7.49 (m, 2H, 2 × H_{tosyl}), 7.23 (d, 2H, *J* = 8.0 Hz, 2 × H_{tosyl}), 6.86 (dd, 1H, *J* = 8.5, 2.1 Hz, H_{benz}), 6.66 (d,1H, *J* = 8.4 Hz, H_{benz}), 4.50 (dd,1H, *J* = 14.6, 3.3 Hz, $H_{oxazine}$), 4.36 - 4.26 (m, 2H, $-CH_2-$), 3.74 - 3.68 (m, 1H, $H_{oxazine}$), 3.17 (dd, 1H, *J* = 14.6, 9.7 Hz, $H_{oxazine}$), 2.34 (s, 3H, CH_{3tosyl}), 2.29 (s, 3H, CH_3). ^{13}C NMR (101 MHz, $CDCl_3$) δ (ppm) 153.1 (C_{purine}), 153.0 (C_{purine}), 151.9 (C_{purine}), 146.6 (CH_{purine}), 144.7 (C_{tosyl}), 143.3 (C_{benz}), 135.1 (C_{tosyl}), 131.4 (C_{purine}), 130.5 (C_{benz}), 130.1 (2 × CH_{tosyl}), 127.3 (CH_{benz}), 127.1 (2 × CH_{tosyl}), 124.5 (CH_{benz}), 122.8 (C_{benz}), 117.0 (CH_{benz}), 69.1 ($CH_{oxazine}$), 45.8 ($-CH_2-$), 45.6 ($CH_{2oxazine}$), 21.5 (CH_{3tosyl}), 20.9 ($CH_{3\ benzo}$). HRMS (ESI) (m/z) calcd for $C_{22}H_{20}N_5O_3SCl_2$ $[M+H]^+$ 504.0664; found 504.0678.

2-((6-Bromo-9*H*-purin-9-yl)methyl)-6-methyl-4-tosyl-3,4-dihydro-2*H*-benzo[b][1,4]oxazine (**13**). White solid; 87% yield; mp: 171 – 172ºC. ^1H NMR (400 MHz, $CDCl_3$) δ (ppm) 8.72 (s, 1H, H_{purine}), 8.17 (s, 1H, H_{purine}), 7.56 (d,1H,*J* = 2.0 Hz, H_{benz}), 7.49 (d, 2H, *J* = 8.0 Hz, 2 × H_{tosyl}), 7.20 (d, 2H, *J* = 8.0 Hz, 2 × H_{tosyl}), 6.87 (dd,1H,*J* = 8.4, 2.1 Hz, H_{benz}), 6.69 (d,1H, *J* = 8.3 Hz, H_{benz}), 4.51 (dd,1H,*J* = 14.7, 3.6 Hz, $H_{oxazine}$), 4.43 - 4.27 (m, 2H, $-CH_2-$), 3.78 - 3.72 (m, 1H, $H_{oxazine}$), 3.14 - 3.08 (m, 1H, $H_{oxazine}$), 2.34 (s, 3H, CH_{3tosyl}), 2.30 (s, 3H, CH_3). ^{13}C NMR (101 MHz, $CDCl_3$) δ (ppm) 152.0 (CH_{purine}), 150.5 (C_{purine}), 145.8 (CH_{purine}), 144.5 (C_{tosyl}), 143.4 (C_{purine}), 143.3 (C_{benz}), 135.3 (C_{tosyl}), 134.0 (C_{purine}), 131.3 (C_{benz}), 130.1 (2 × CH_{tosyl}), 127.3 (CH_{benz}), 127.1 (2 × CH_{tosyl}), 124.6 (CH_{benz}), 122.9 (C_{benz}), 117.0 (CH_{benz}), 69.3 ($CH_{oxazine}$), 45.9 ($-CH_2-$), 45.4 ($CH_{2oxazine}$), 21.6 (CH_{3tosyl}), 20.9 (CH_3). HRMS (ESI) (m/z) calcd for $C_{22}H_{21}N_5O_3SBr$ $[M+H]^+$ 514.0548; found 514.0527.

Substituted 4-tosyl-2-((6-(trifluoromethyl)-9*H*-purin-9-yl)methyl)-3,4-dihydro-2H-benzo[b][1,4]oxazine derivatives (compounds 6, 10, 14)

7-Chloro-4-tosyl-2-((6-(trifluoromethyl)-9H-purin-9-yl)methyl)-3,4-dihydro-2*H*-benzo[b][1,4]oxazine (**6**). Brown oil; 80% yield. ^1H NMR (500 MHz, $CDCl_3$) δ (ppm) 9.12 (s, 1H, H_{purine}), 8.27 (s, 1H, H_{purine}), 7.71 (d,1H, *J* = 8.9 Hz, H_{benz}), 7.50 (d, 2H, *J* = 8.4 Hz, 2 × H_{tosyl}), 7.33 - 7.15 (m, 2H, 2 × H_{tosyl}) ,6.95 (dd, 1H, *J* = 8.9, 2.4Hz, H_{benz}), 6.83 (d, 1H, *J* = 2.4 Hz, H_{benz}), 4.58 (dd, 1H, *J* = 14.5, 3.5 Hz, $H_{oxazine}$), 4.46 - 4.38 (m, 2H, $-CH_2-$), 3.83 - 3.78 (m,1H, $H_{oxazine}$), 3.11 (dd,1H, *J* = 14.5, 9.7 Hz, $H_{oxazine}$), 2.36 (s, 3H, $-CH_3$). ^{13}C NMR (126 MHz, $CDCl_3$) δ (ppm) 153.7 (C_{purine}),

152.1 (CH_{purine}), 147.7 (CH_{purine}), 146.0 (C_{benz}), 145.4 (q, *J* = 37.6 Hz, C_{purine}), 144.9 (C_{tosyl}), 134.9 (C_{tosyl}), 131.6 (C_{benz}), 130.2 (2 × CH_{tosyl}), 129.8 (C_{purine}), 127.1 (2 × CH_{tosyl}), 125.4 (CH_{benz}), 124.0 (q, *J* = 275.9 Hz, CF_3), 122.1 (CH_{benz}), 122.0 (C_{benz}), 117.5 (CH_{benz}), 69.7 ($CH_{oxazine}$), 45.6 ($CH_{2oxazine}$), 45.0 ($-CH_2$), 21.6 (CH_{3tosyl}). HRMS (ESI) (m/z) calcd for $[M+Cl]^-$ $C_{22}H_{17}N_5O_3SCl_2F_3$ 558.0381; found 558.0377.

6-Bromo-4-tosyl-2-((6-(trifluoromethyl)-9*H*-purin-9-yl)methyl)-3,4-dihydro-2*H*-benzo[b][1,4]oxazine (**10**). Brown solid; 84% yield; mp: 110 – 111ºC. ^1H NMR (400 MHz, $CDCl_3$) δ 9.13 (s, 1H, H_{purine}), 8.30 (s, 1H, H_{purine}), 7.95 (d,1H, *J* = 2.3 Hz, H_{benz}), 7.61 - 7.50 (m, 2H, 2 × H_{tosyl}), 7.25 (d, 2H, *J* = 8.1 Hz, 2 × H_{tosyl}), 7.19 (dd,1H, *J* = 8.8, 2.3 Hz, H_{benz}), 6.70 (d,1H, *J* = 8.8 Hz, H_{benz}), 4.61 (dd, 1H, *J* = 14.8, 3.4 Hz, $H_{oxazine}$), 4.48 - 4.36 (m, 2H, $-CH_2-$), 3.90 – 3.82 (m, 1H, $H_{oxazine}$), 3.14 (dd, 1H, *J* = 14.8, 9.6 Hz, $H_{oxazine}$), 2.37 (s, 3H, CH_{3tosyl}).^{13}C NMR (101 MHz, $CDCl_3$) δ (ppm) 153.7 (C_{purine}), 152.1 (CH_{purine}), 147.8 (CH_{purine}), 145.4 (q, *J* = 37.6 Hz, C_{purine}), 145.0 (C_{tosyl}), 144.5 (C_{benz}), 134.8 (C_{tosyl}), 130.2 (2 × CH_{tosyl}), 129.8 (C_{purine}), 129.4 (CH_{benz}), 127.2 (2 × CH_{tosyl}), 126.7 (CH_{benz}), 124.5 (C_{benz}),123.0 (q, *J* = 260.0 Hz, CF_3), 118.9 (CH_{benz}), 113.8 (C_{benz}), 69.7 ($CH_{oxazine}$), 45.5 ($CH_{2oxazine}$), 45.0 ($-CH_2$), 21.6 (CH_{3tosyl}). HRMS (ESI) (m/z) calcd for $[M+H]^+$ $C_{22}H_{18}N_5O_3F_3SBr$ 568.0266; found 568.0235.

6-Methyl-4-tosyl-2-((6-(trifluoromethyl)-9*H*-purin-9-yl)methyl)-3,4-dihydro-2*H*-benzo[b][1,4]oxazine (**14**). Brown solid; 86% yield; mp: 168 – 169ºC.^1H NMR (400 MHz, $CDCl_3$) δ (ppm) 9.14 (s, 1H, H_{purine}), 8.43 (s, 1H_{purine}), 7.58 (dd,1H,*J* = 2.1, 0.8 Hz, H_{benz}), 7.53 (d, 2H, *J* = 8.0 Hz, 2 × H_{tosyl}), 7.22 (d, 2H, *J* = 7.8 Hz, 2 × H_{tosyl}), 6.89 (dd,1H, *J* = 8.2, 2.0 Hz, H_{benz}), 6.71 (d, 1H, *J* = 8.3 Hz, H_{benz}), 4.61 (d, 1H, *J* = 13.7 Hz, $H_{oxazine}$), 4.45 - 4.35 (m, 2H, $-CH_2-$), 3.90 - 3.82 (m, 1H, $H_{oxazine}$), 3.16 (dd, 1H, *J* = 14.0, 9.2 Hz, $H_{oxazine}$), 2.36 (s, 3H, CH_{3tosyl}), 2.31 (s, 3H, CH_3). ^{13}C NMR (101 MHz, $CDCl_3$) δ (ppm) 152.1 (CH_{purine}), 147.7 (C_{purine}), 145.4 (q, *J* = 37.7 Hz, C_{purine}), 144.6 (CH_{purine}), 143.3 (C_{benz}), 135.3 (C_{tosyl}), 131.5 (C_{benz}), 130.1 (2 × $CH_{tosyl,}$ C_{purine}), 127.4 (CH_{benz}), 127.2 (2 × CH_{tosyl}), 124.5 (CH_{benz}), 122.9 (C_{benz}), 120.8 (q, *J* = 274.9 Hz, CF_3), 117.1 (CH_{benz}), 69.4 ($CH_{oxazine}$), 46.0 ($-CH_2-$), 45.4 ($CH_{2oxazine}$) 21.6 (CH_{3tosyl}), 20.9 (CH_3). HRMS (ESI) (m/z) calcd for $C_{23}H_{21}N_5O_3F_3S$ $[M+H]^+$ 504.1317; found 504.1337.

1.2. NMR Spectra

(7-Chloro-3,4-dihydro-2*H*-benzo[b][1,4]oxazin-2-yl)methanol (**18**)

^{1}H NMR

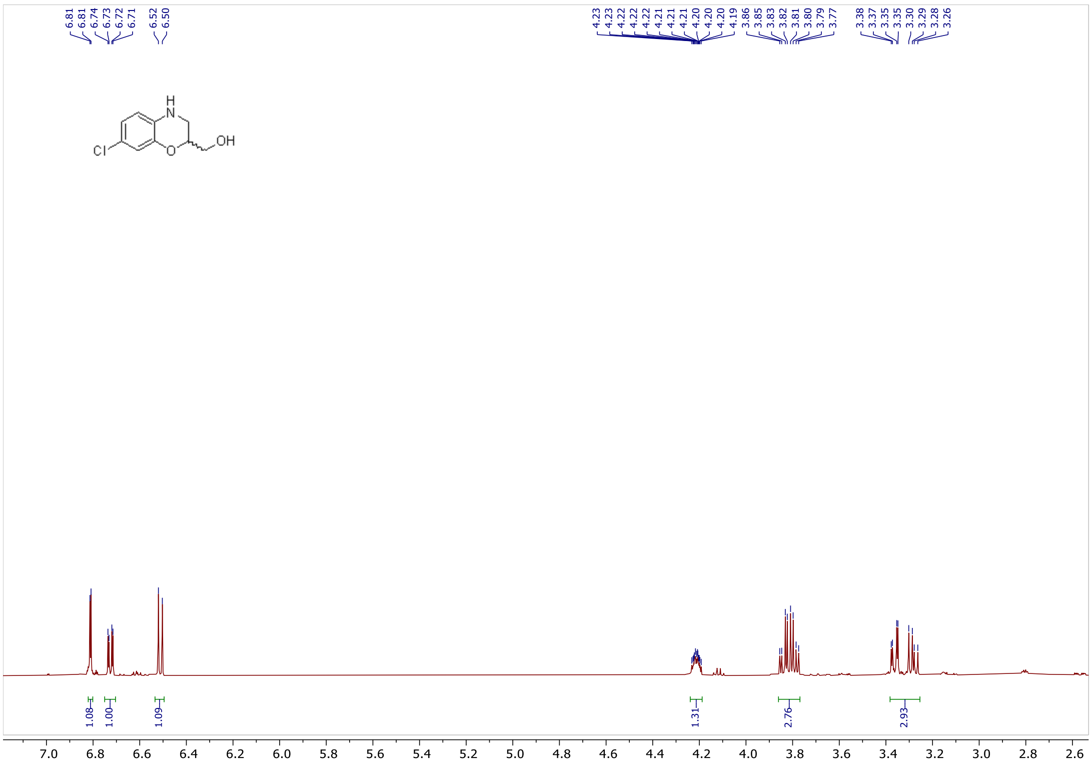

^{13}C NMR

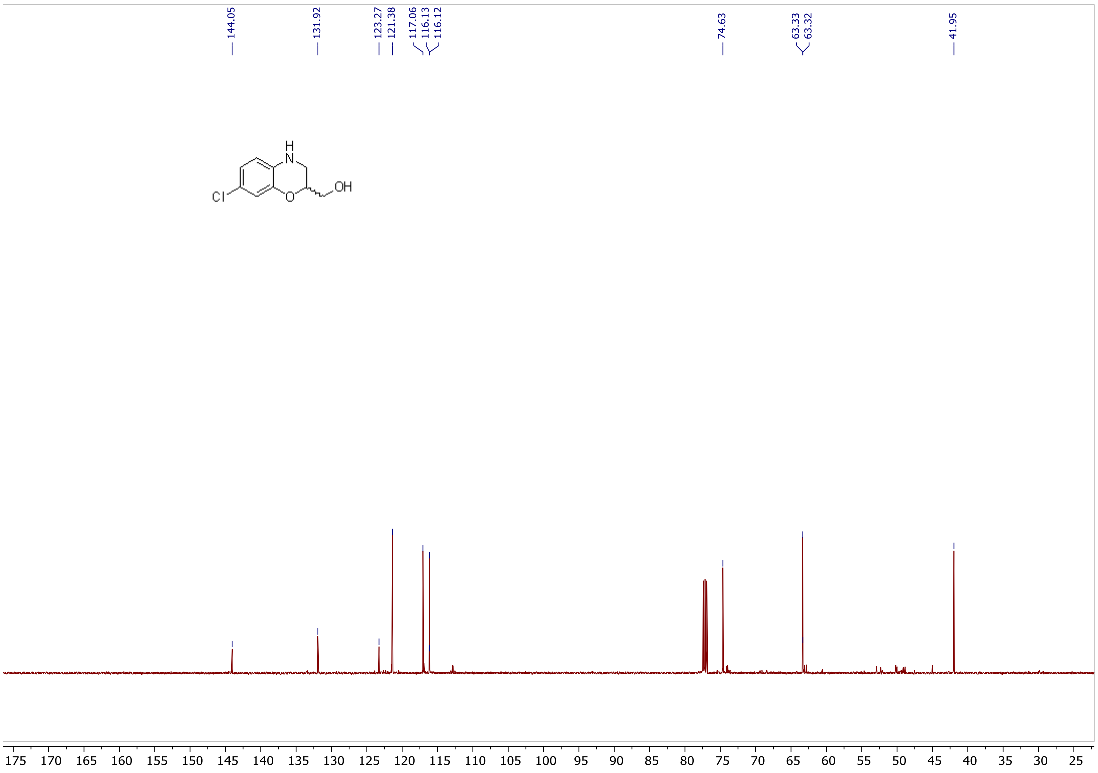

(6-Bromo-3,4-dihydro-2*H*-benzo[b][1,4]oxazin-2-yl)methanol (**19**)

^{1}H NMR

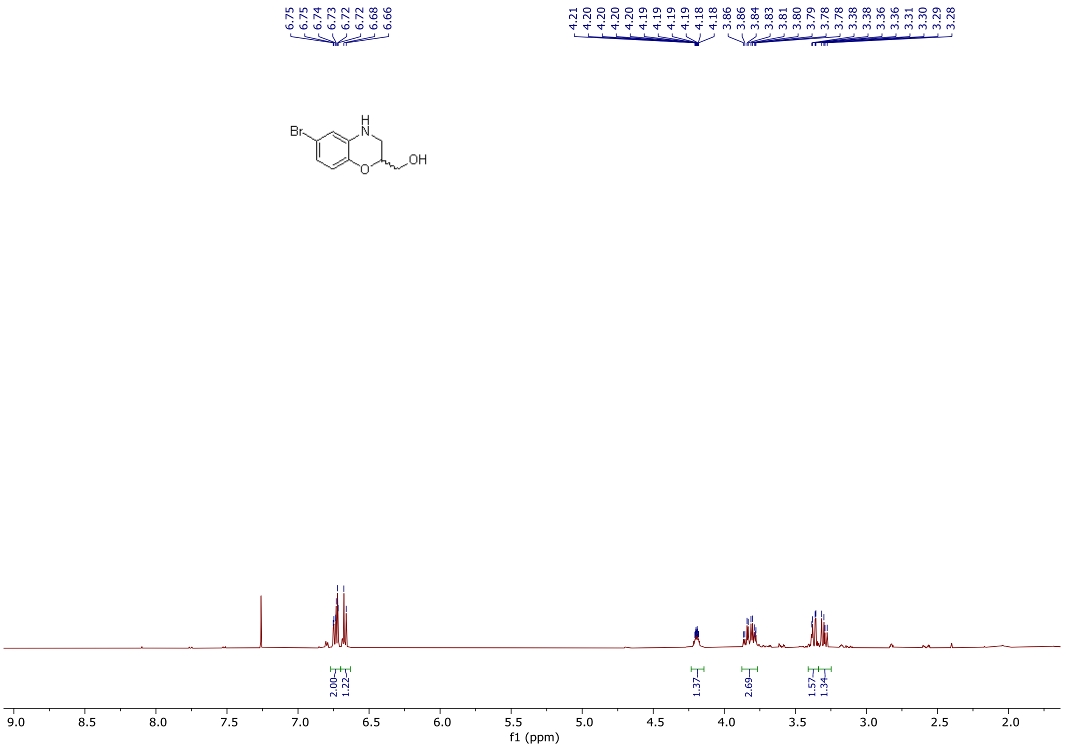

^{13}C NMR

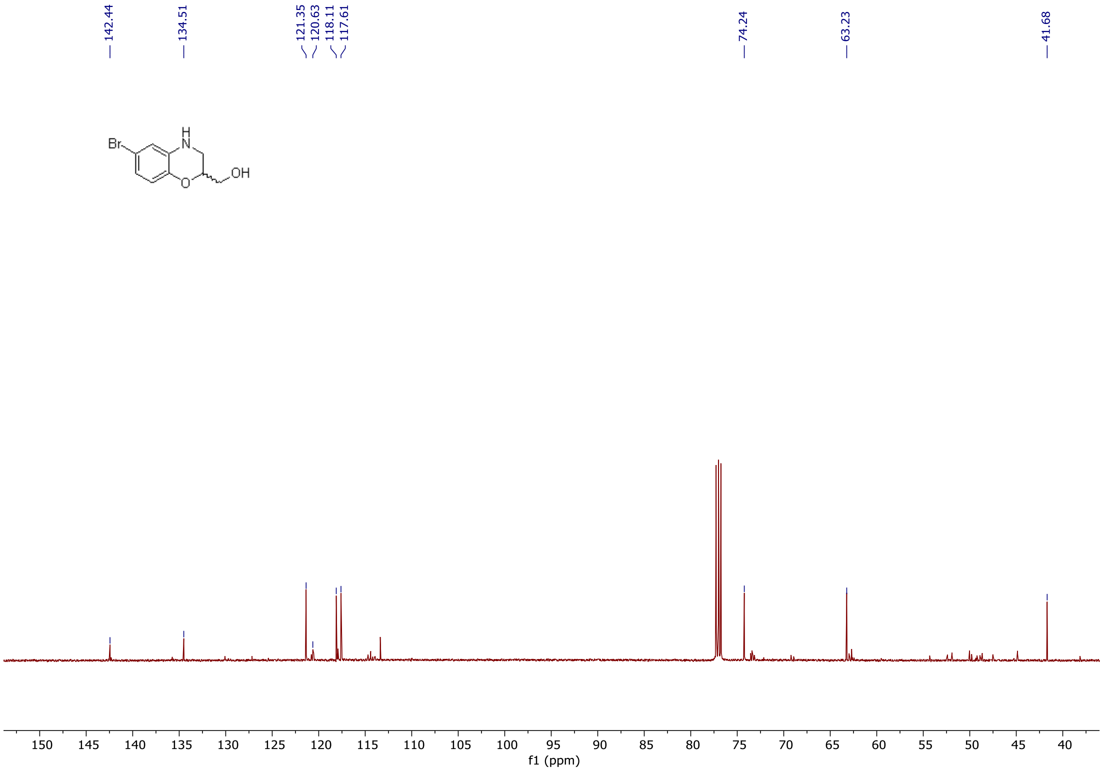

(6-Methyl-3,4-dihydro-2*H*-benzo[b][1,4]oxazin-2-yl)methanol (**20**).

^{1}H NMR

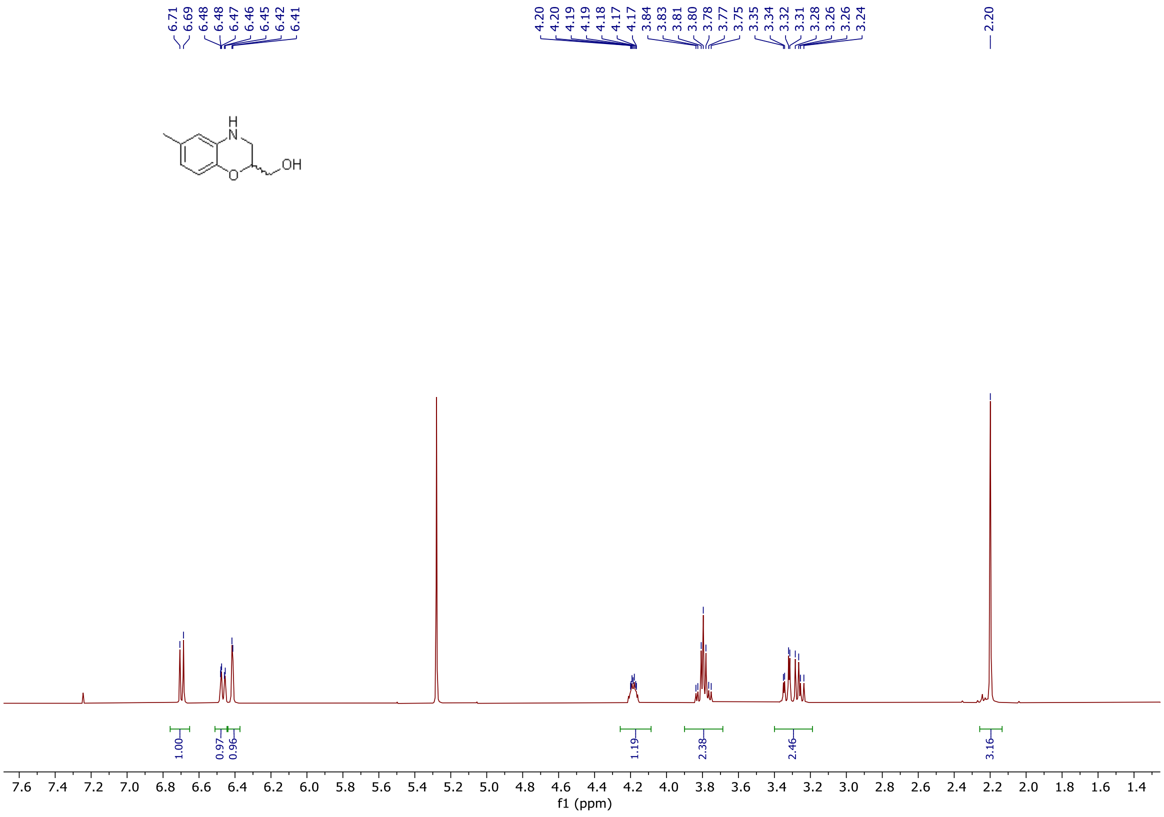

^{13}C NMR

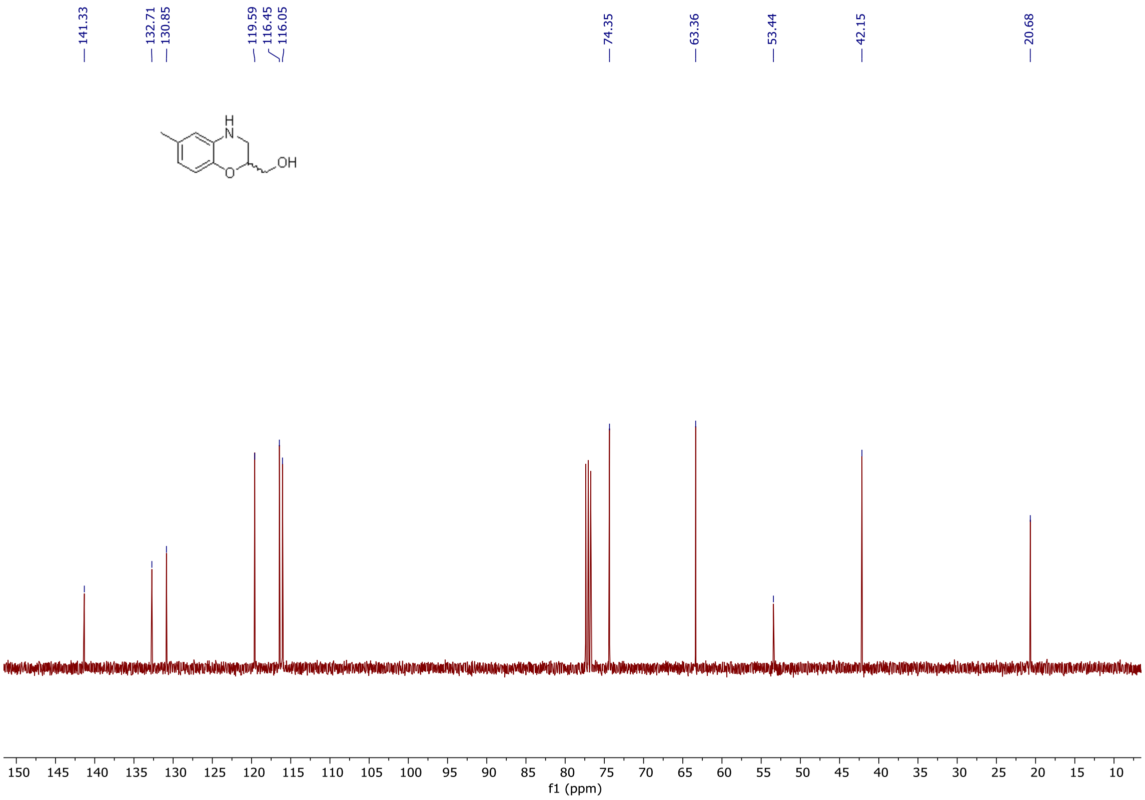

(7-Chloro-4-tosyl-3,4-dihydro-2*H*-benzo[b][1,4-]oxazin-2-yl)metanol (**21**).

^{1}H NMR

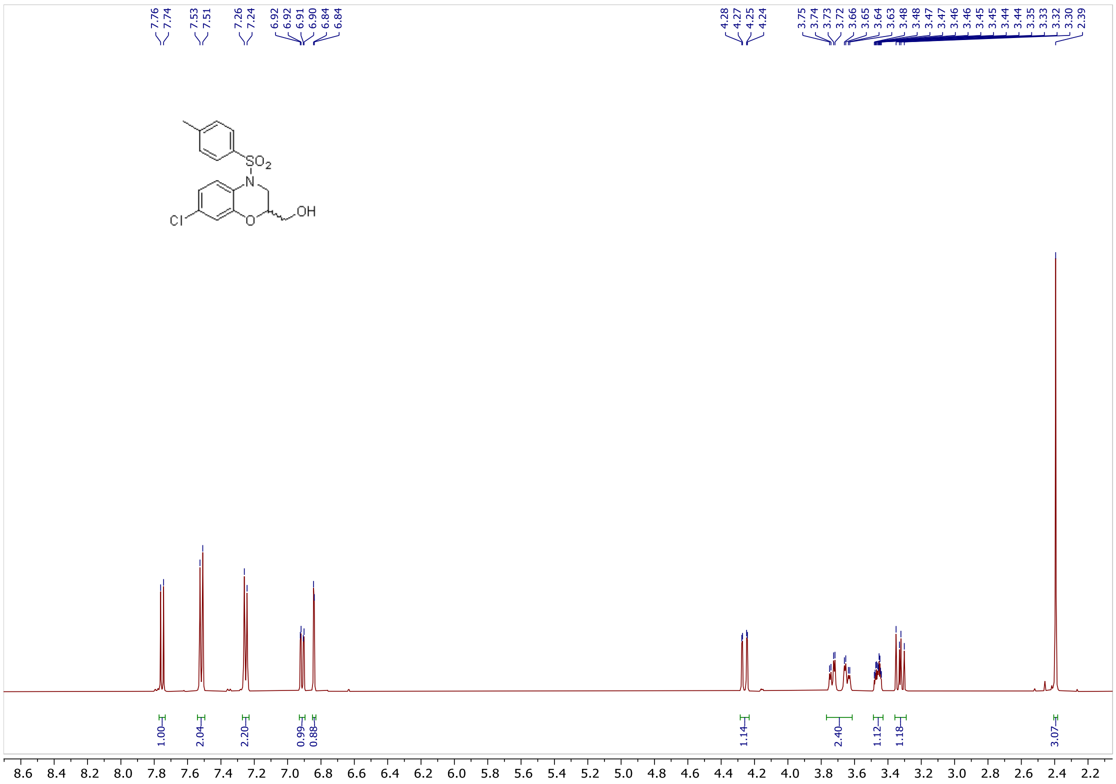

^{13}C NMR

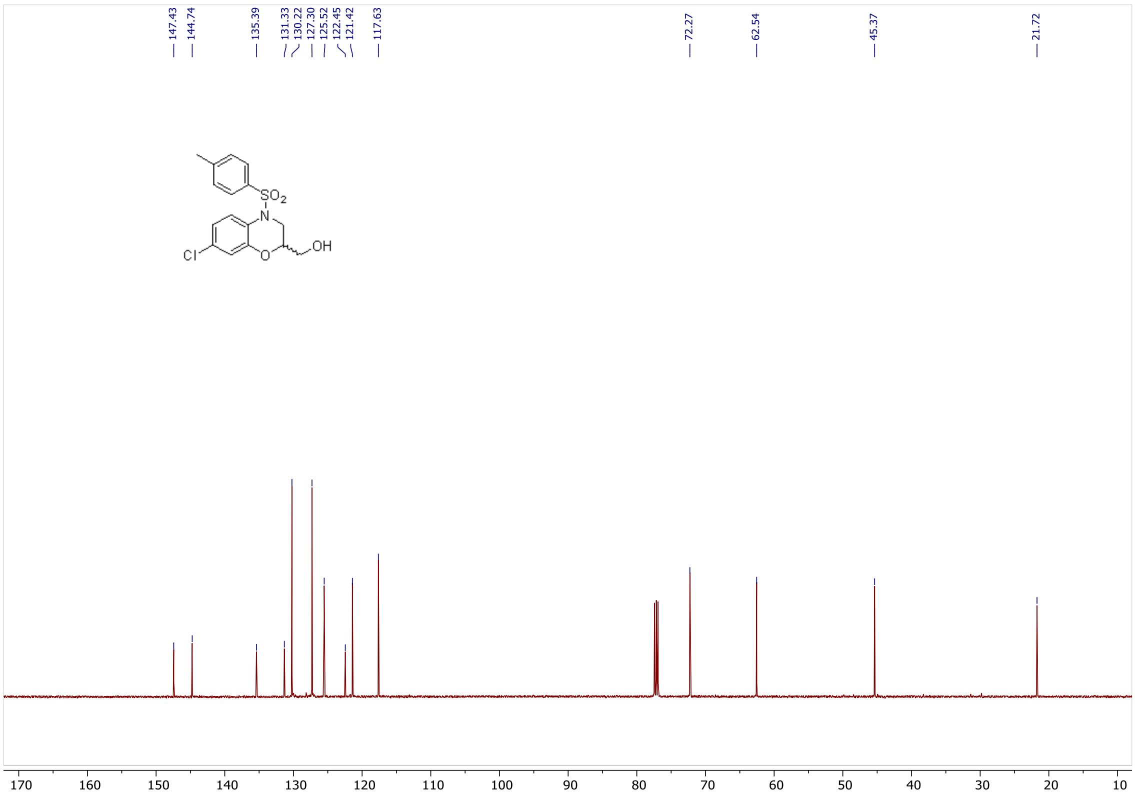

(6-Bromo-4-tosyl-3,4-dihydro-2*H*-benzo[b][1,4-]oxazin-2-yl)methanol (**22**).

^{1}H NMR

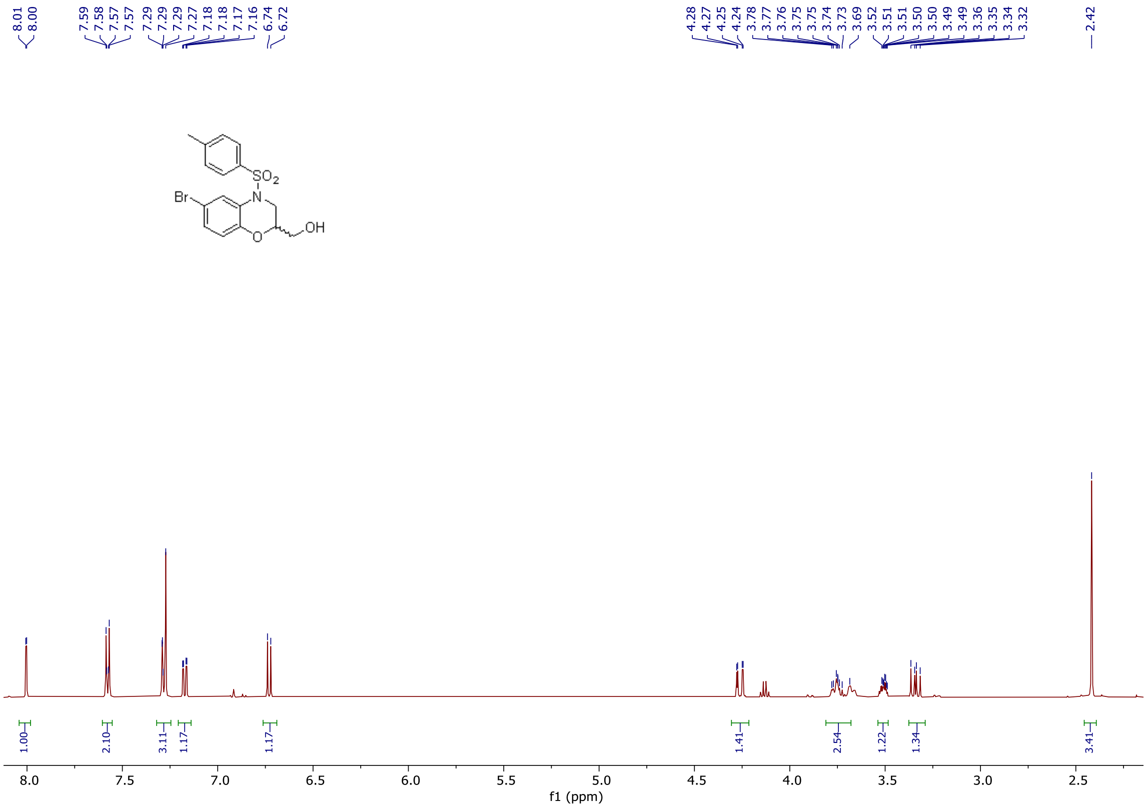

^{13}C NMR

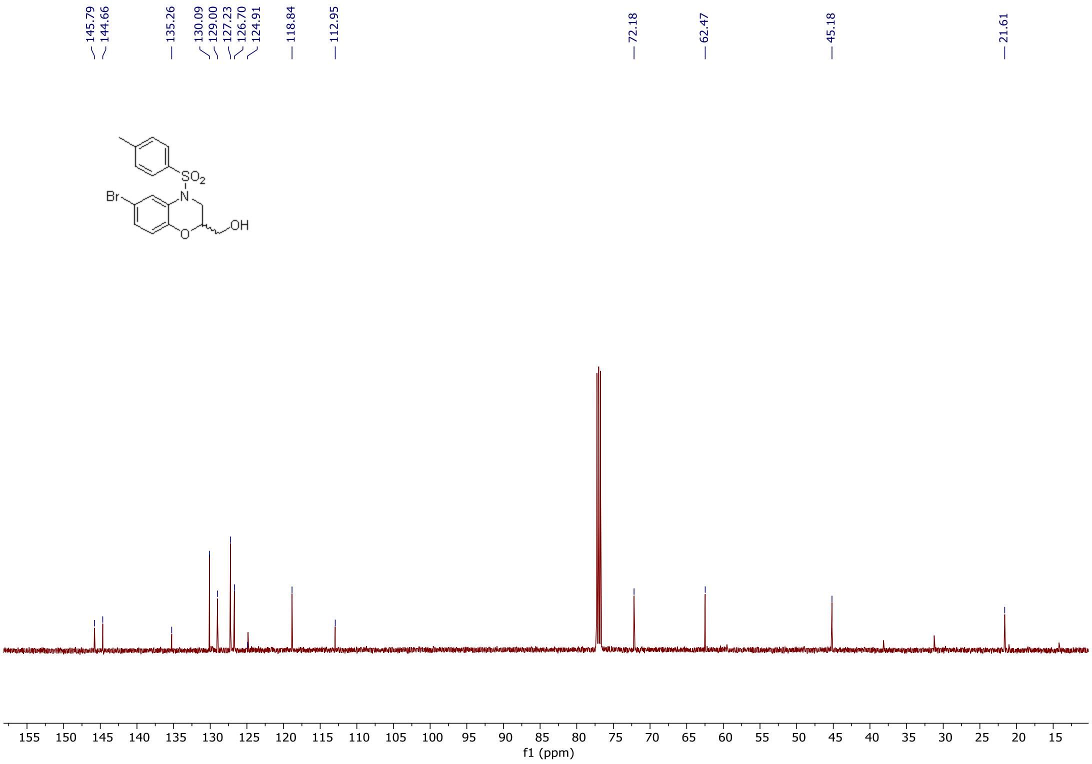

(6-Methyl-4-tosyl-3,4-dihydro-2*H*-benzo[b][1,4-]oxazin-2-yl)methanol (**23**)

^{1}H NMR

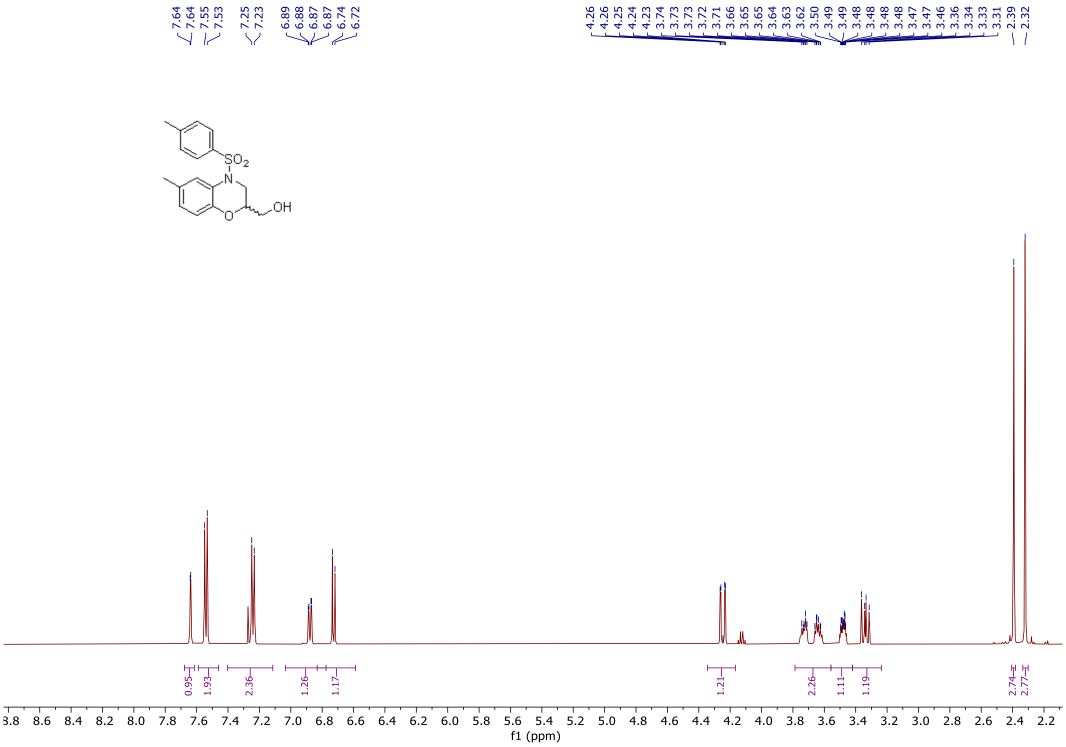

^{13}C NMR

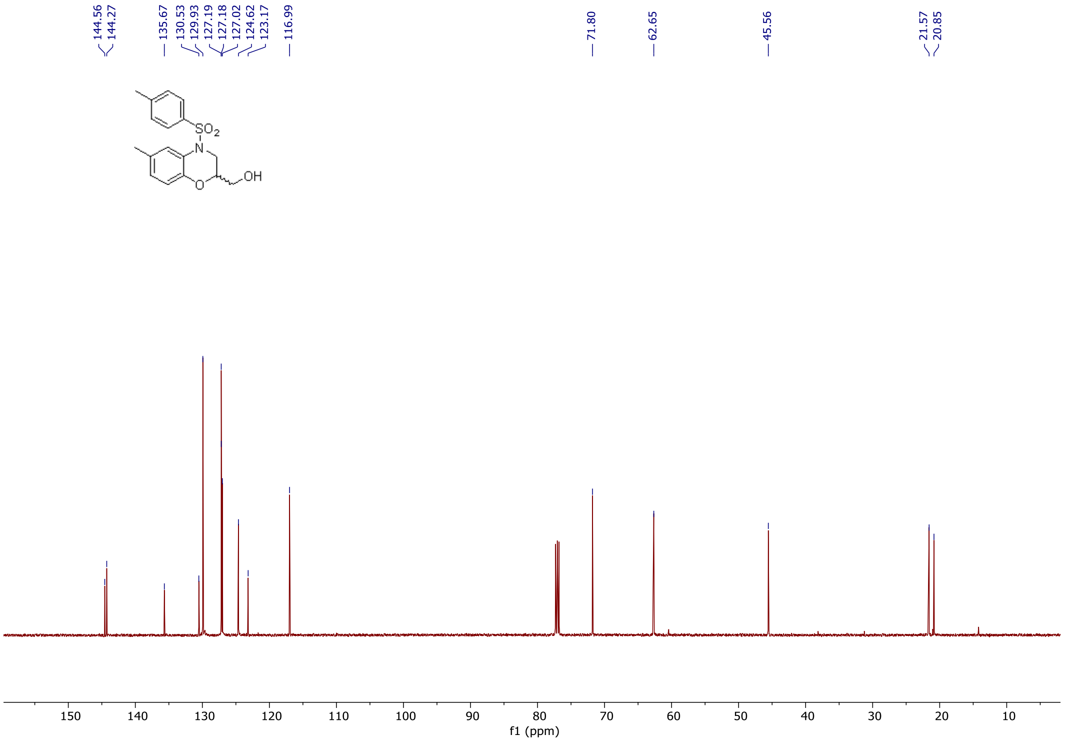

7-Chloro-2-((6-chloro-9*H*-purin-9-yl)methyl)-4-tosyl-3,4-dihydro-2*H*-benzo[b][1,4]oxazine (**3**).

^{1}H NMR

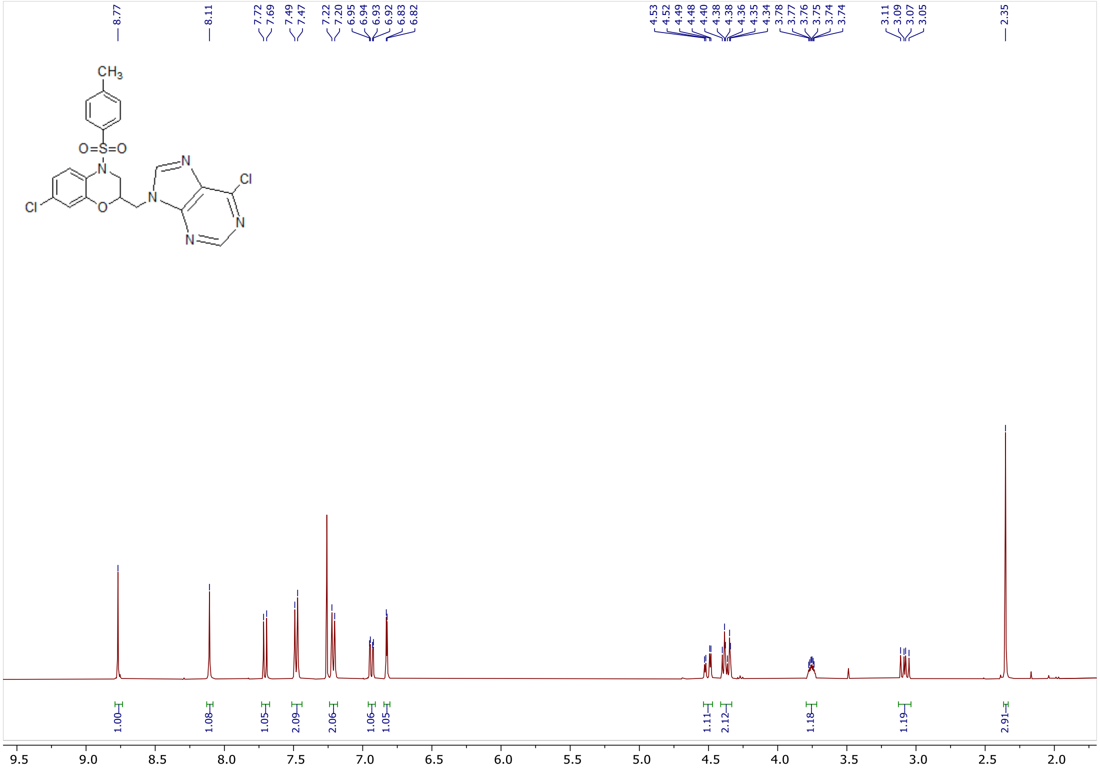

^{13}C NMR

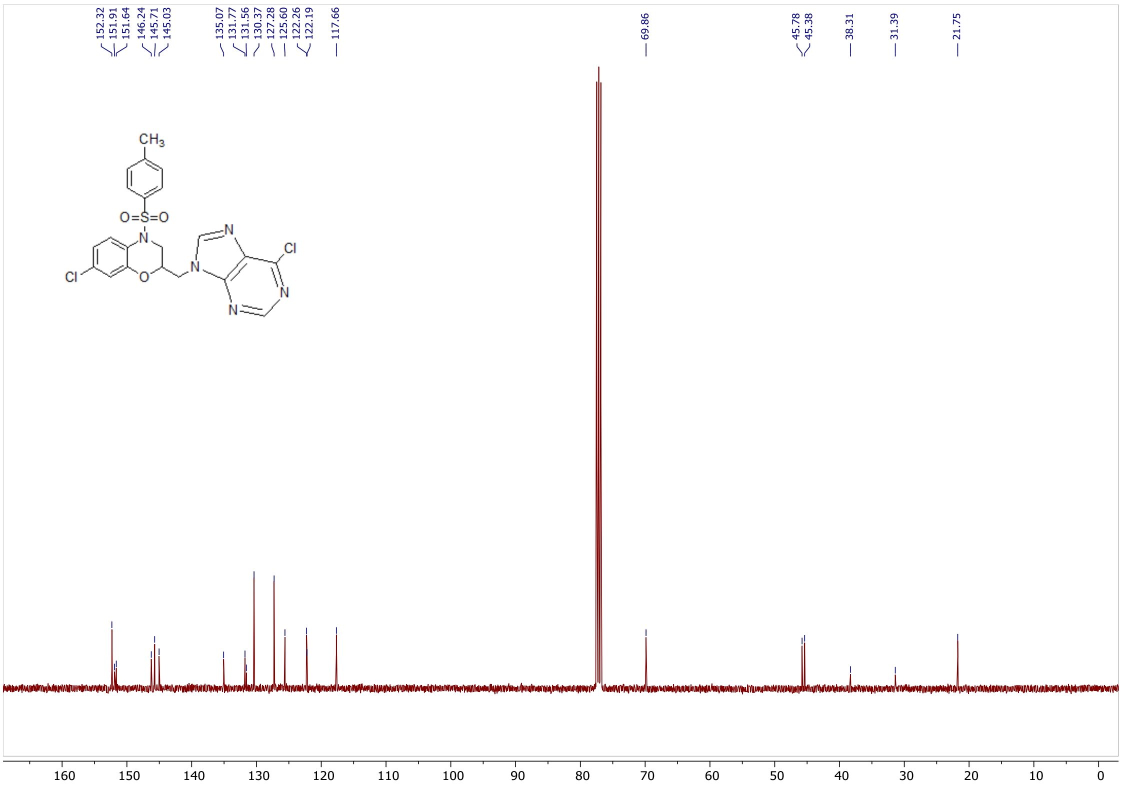

7-Chloro-2-((2,6-dichloro-9*H*-purin-9-yl)methyl)-4-tosyl-3,4-dihydro-2*H*-benzo[b][1,4]oxazine (**4**)

^{1}H NMR

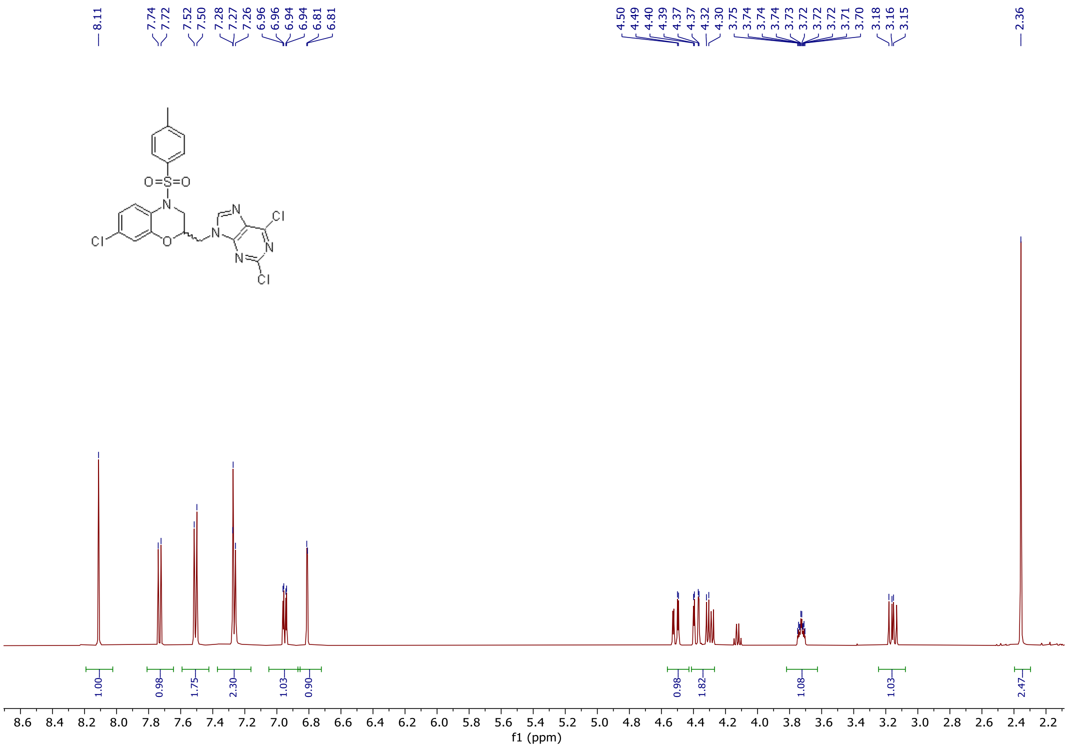

^{13}C NMR

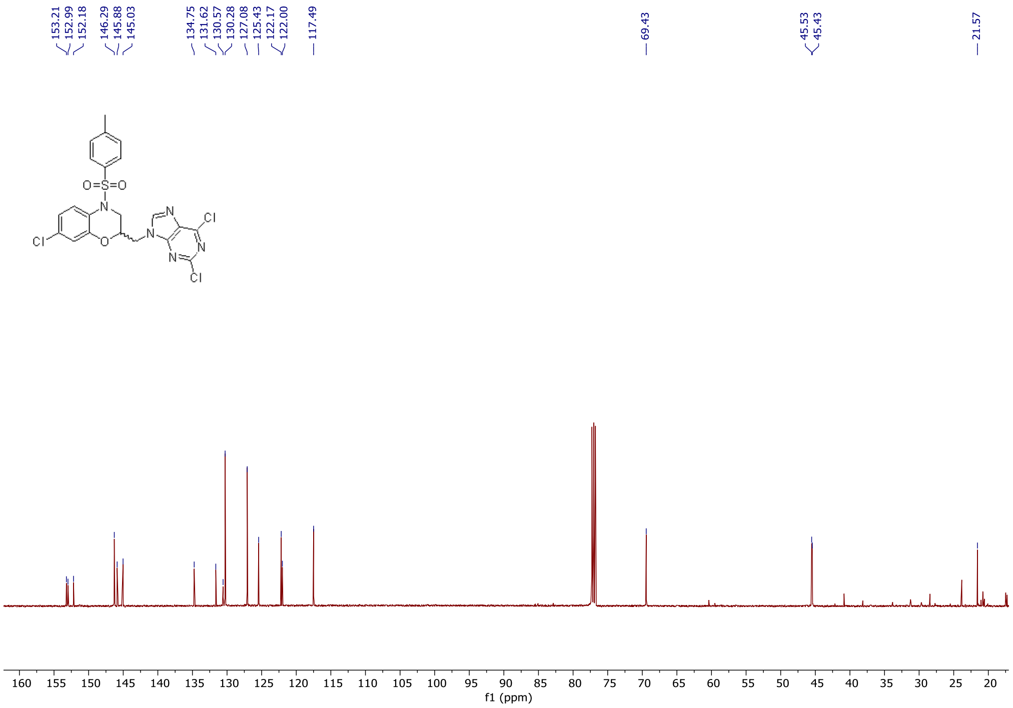

7-Chloro-2-((6-bromo-9*H*-purin-9-yl)methyl)-4-tosyl-3,4-dihydro-2*H*-benzo[b][1,4]oxazine (**5**).

^{1}H NMR

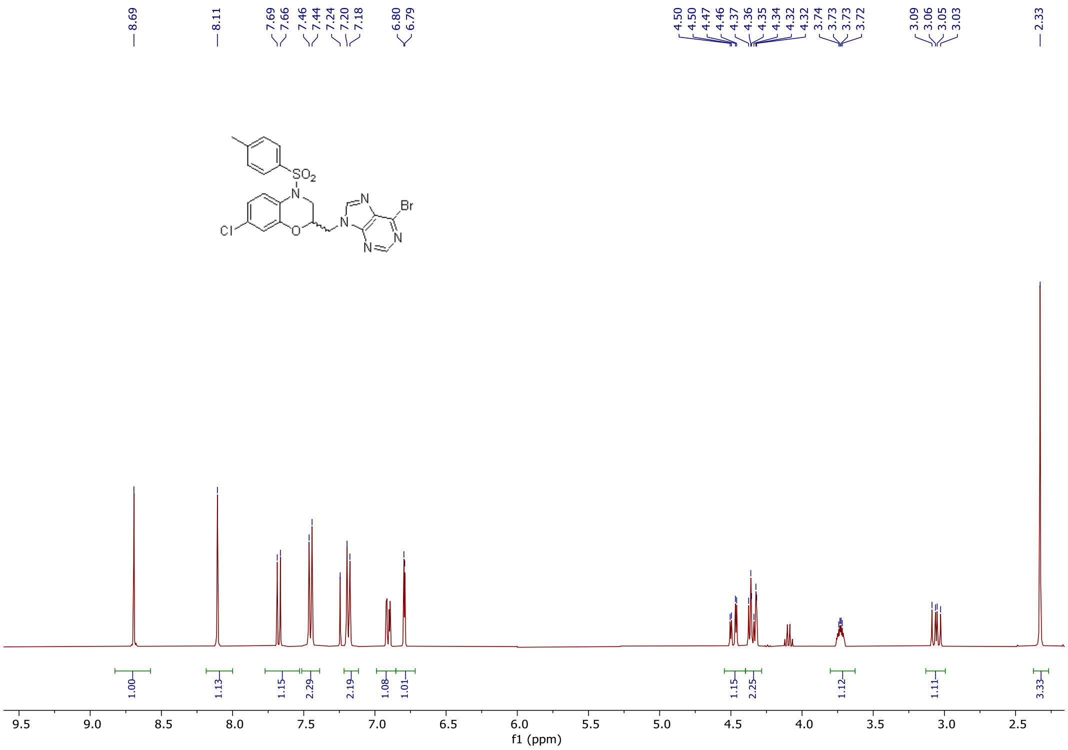

^{13}C NMR

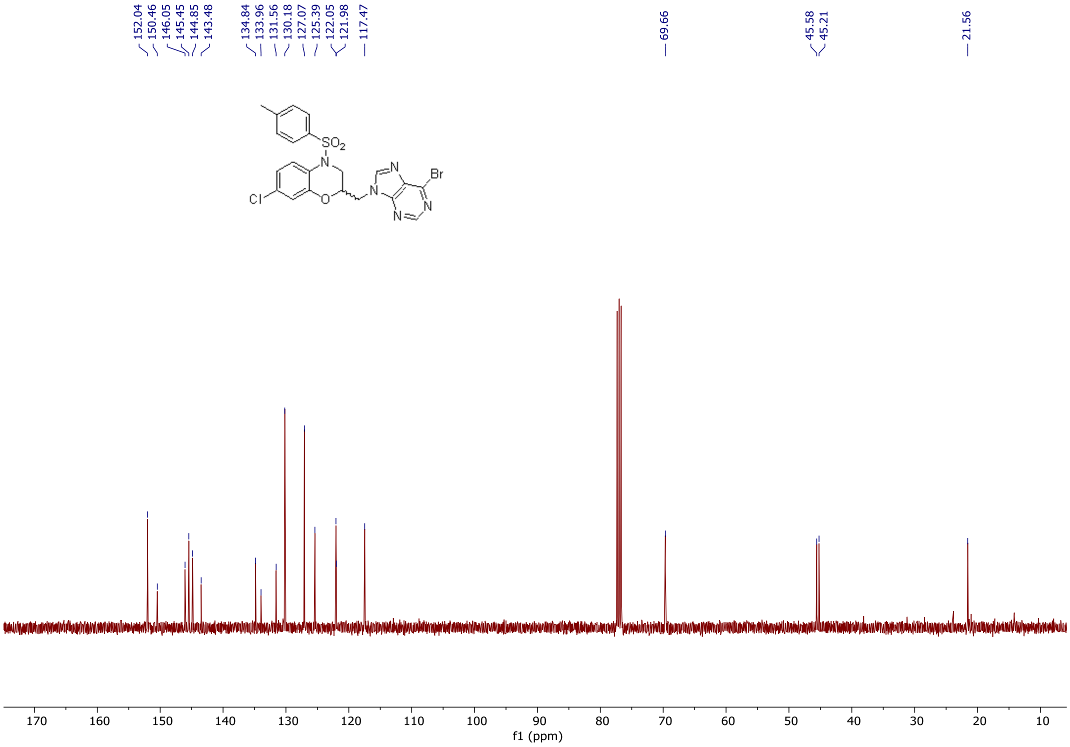

7-Chloro-4-tosyl-2-((6-(trifluoromethyl)-9*H*-purin-9-yl)methyl)-3,4-dihydro-2*H*-benzo[b][1,4]oxazine (**6**).

^{1}H NMR

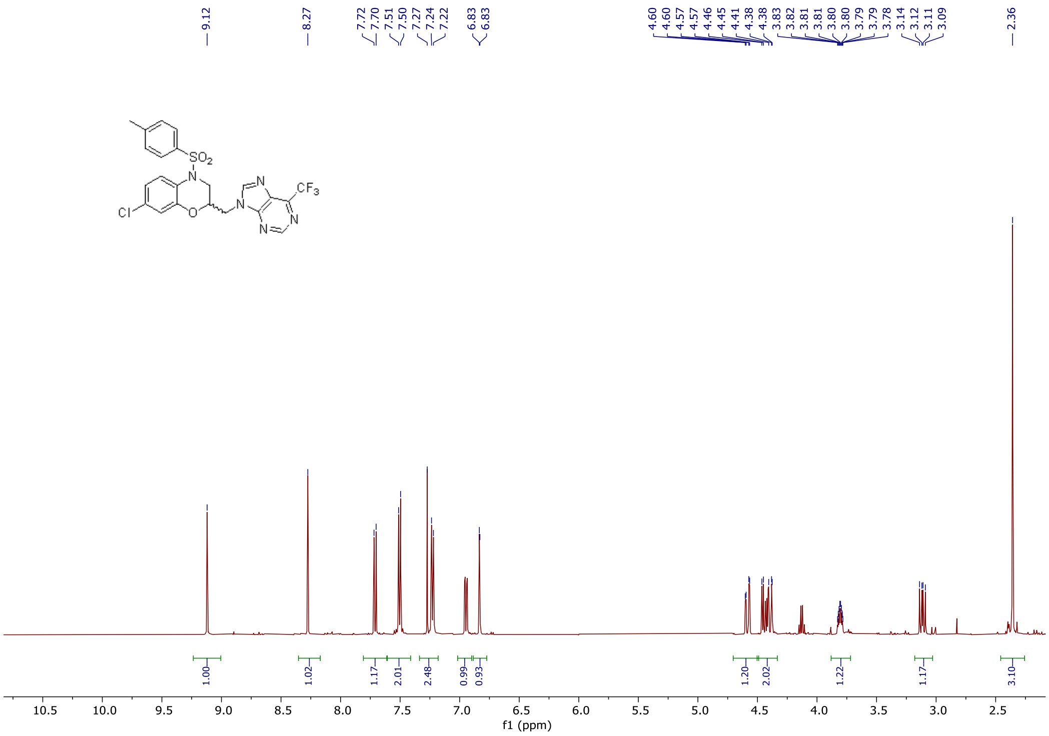

^{13}C NMR

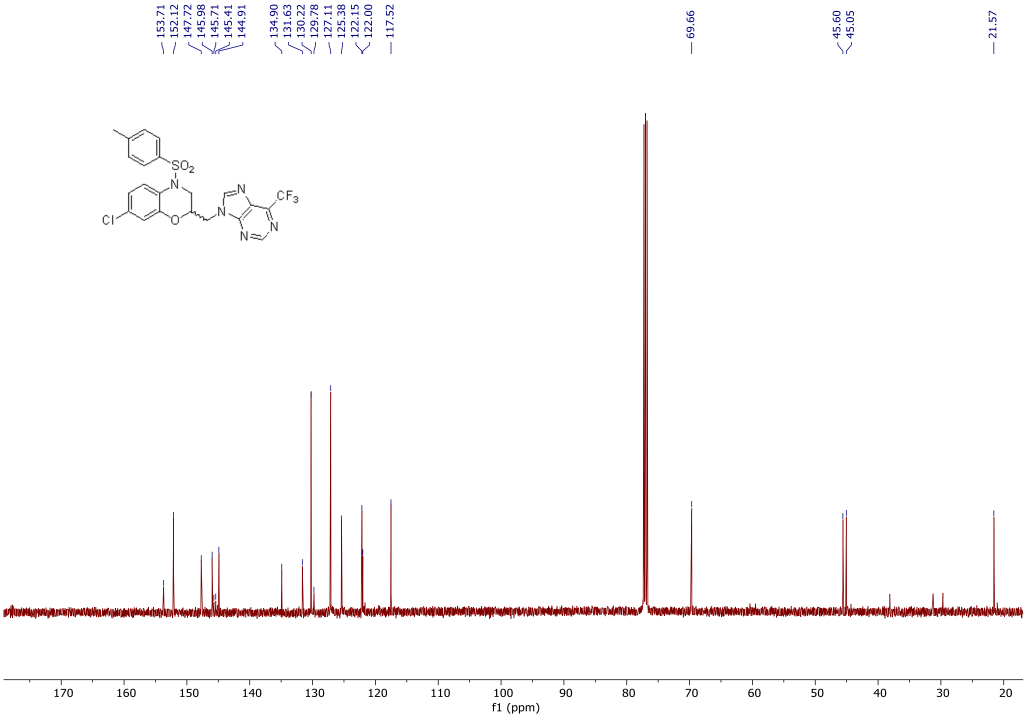

6-Bromo-2-((6-chloro-9*H*-purin-9-yl)methyl)-4-tosyl-3,4-dihydro-2*H*-benzo[b][1,4]oxazine (**7**)

^{1}H NMR

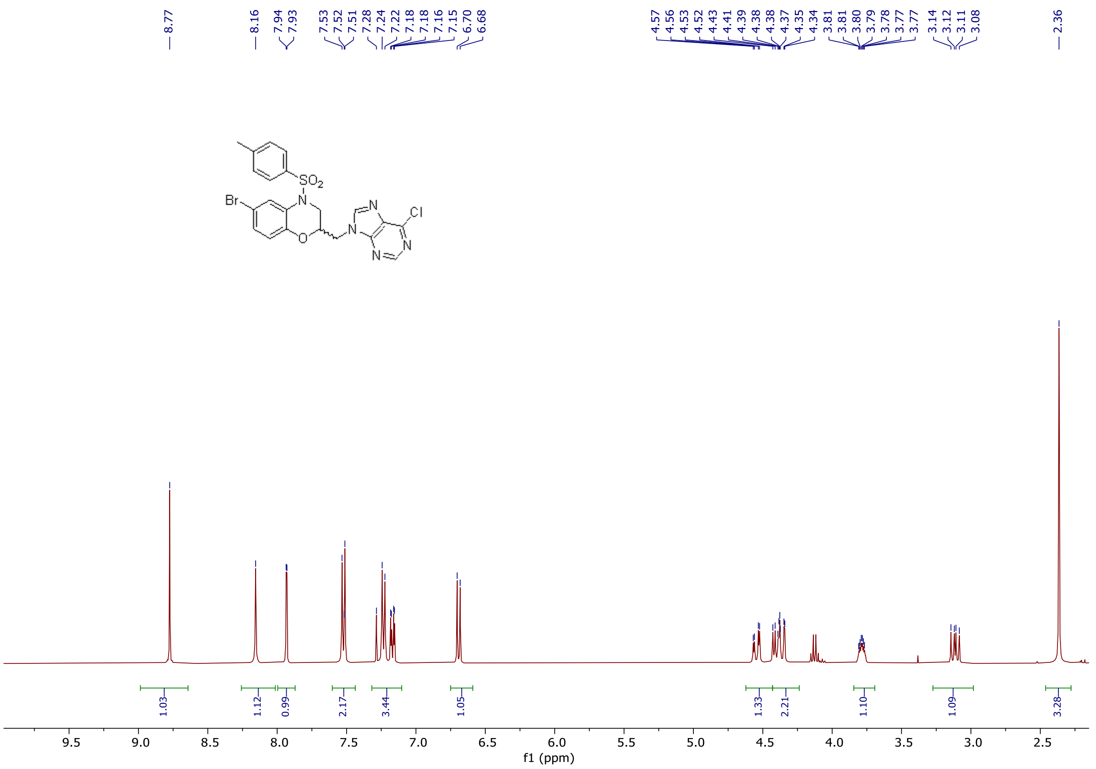

^{13}C NMR

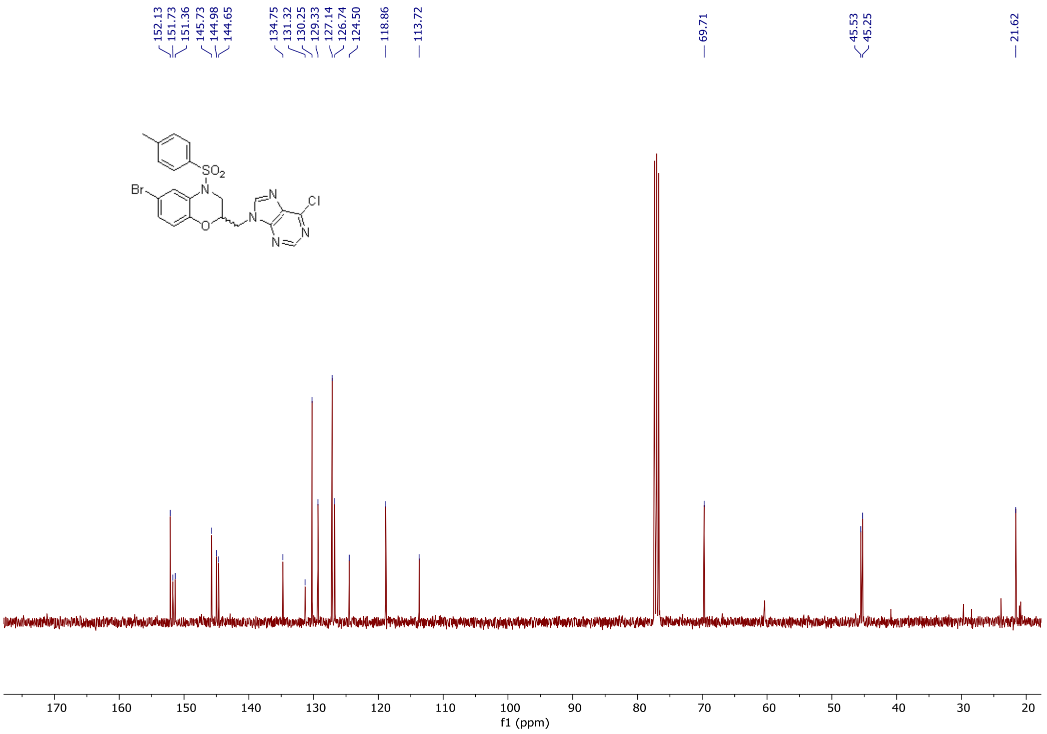

6-Bromo-2-((2,6-dichloro-9*H*-purin-9-yl)methyl)-4-tosyl-3,4-dihydro-2*H*-benzo[b][1,4]oxazine (**8**)

^{1}H NMR

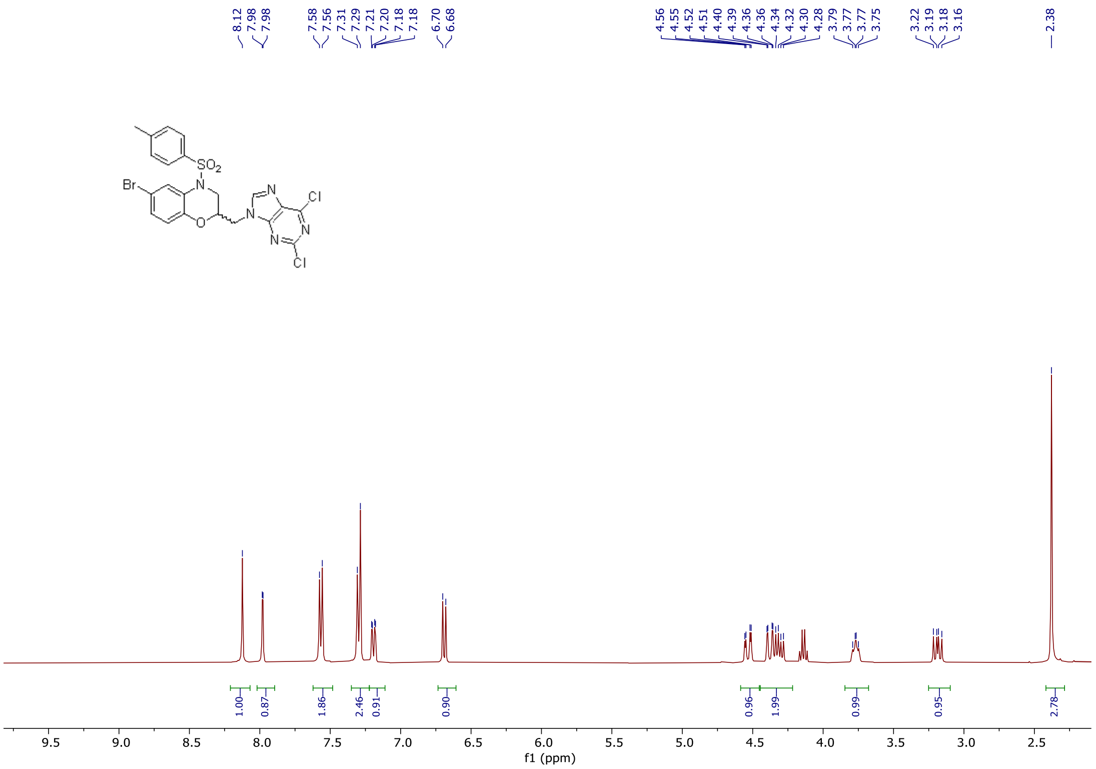

^{13}C NMR

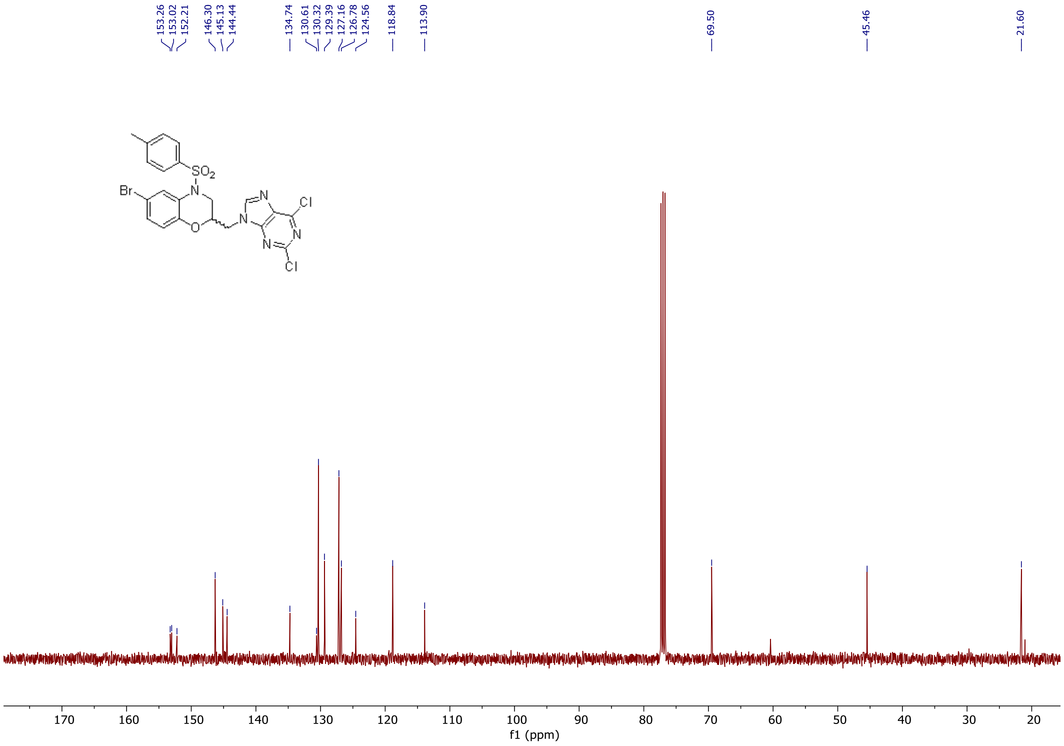

6-Bromo-2-((6-bromo-9*H*-purin-9-yl)methyl)-4-tosyl-3,4-dihydro-2*H*-benzo[b][1,4]oxazine (**9**)

^{1}H NMR

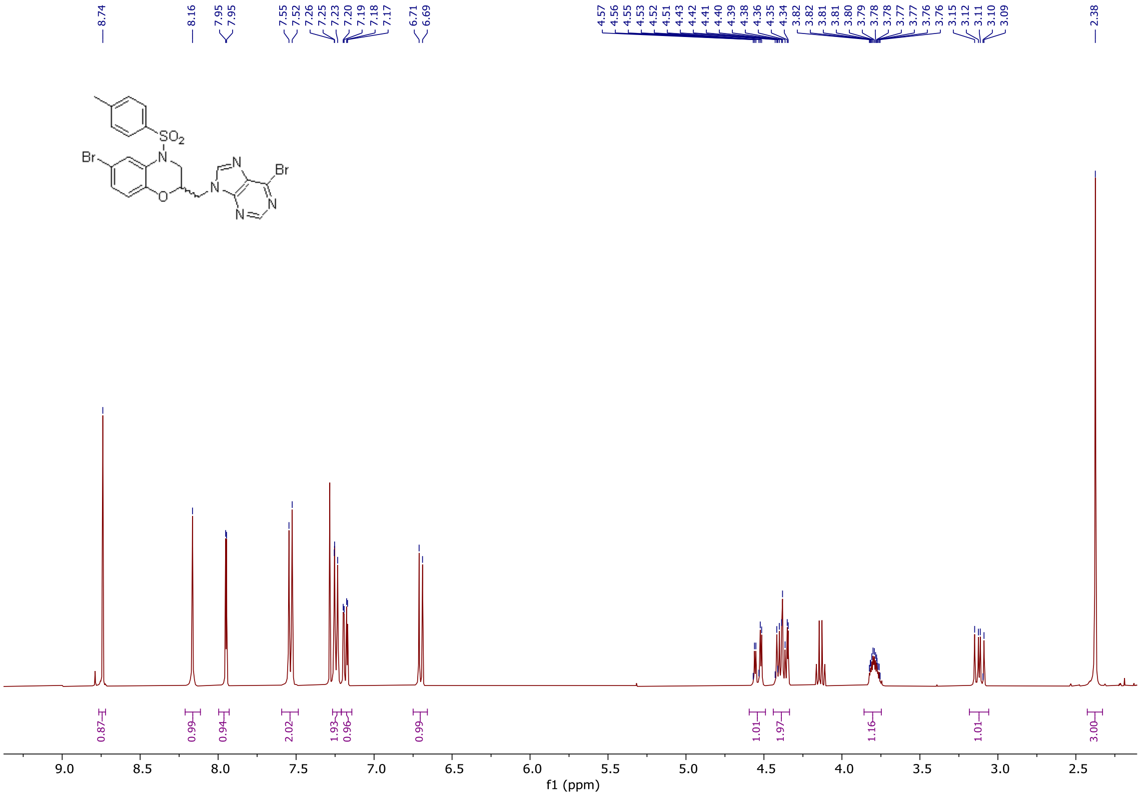

^{13}C NMR

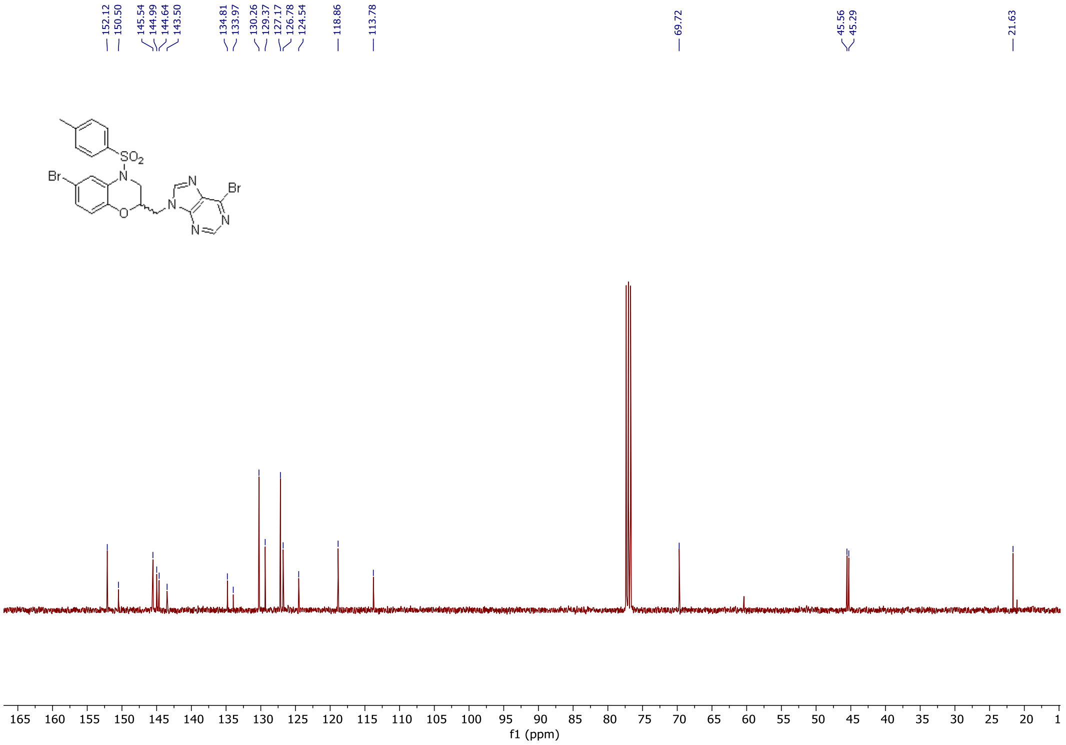

6-Bromo-4-tosyl-2-((6-(trifluoromethyl)-9*H*-purin-9-yl)methyl)-3,4-dihydro-2*H*-benzo[b][1,4]oxazine (**10**)

^{1}H NMR

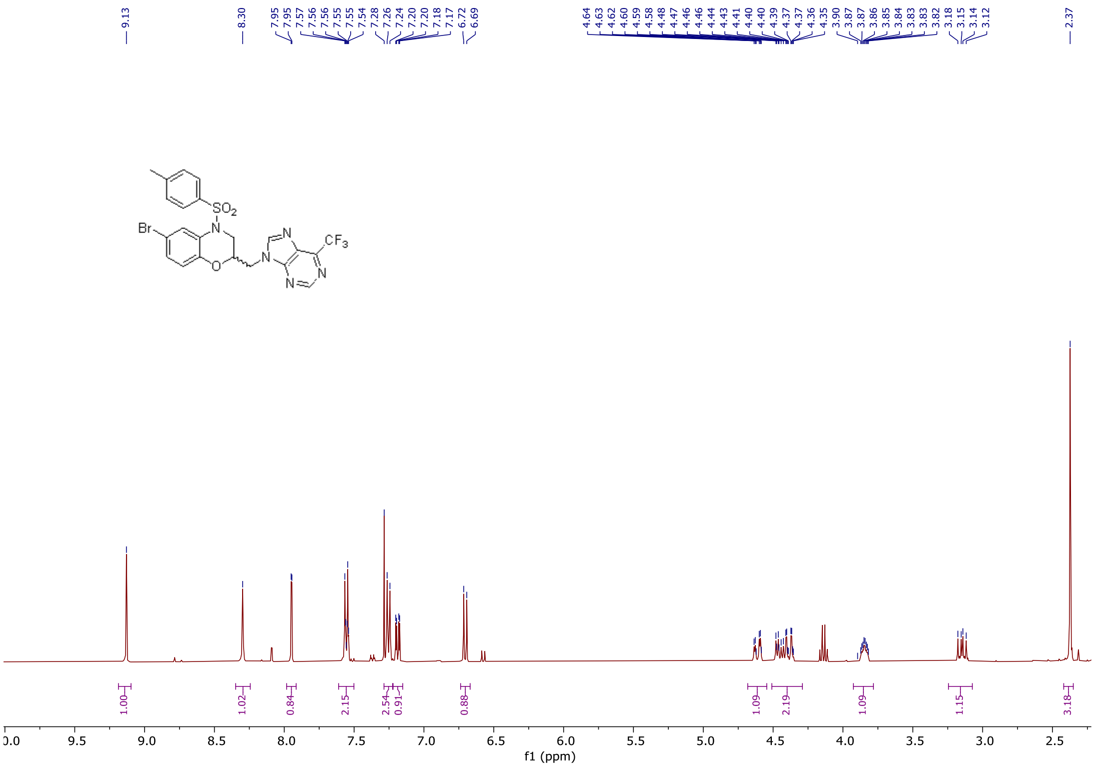

^{13}C NMR

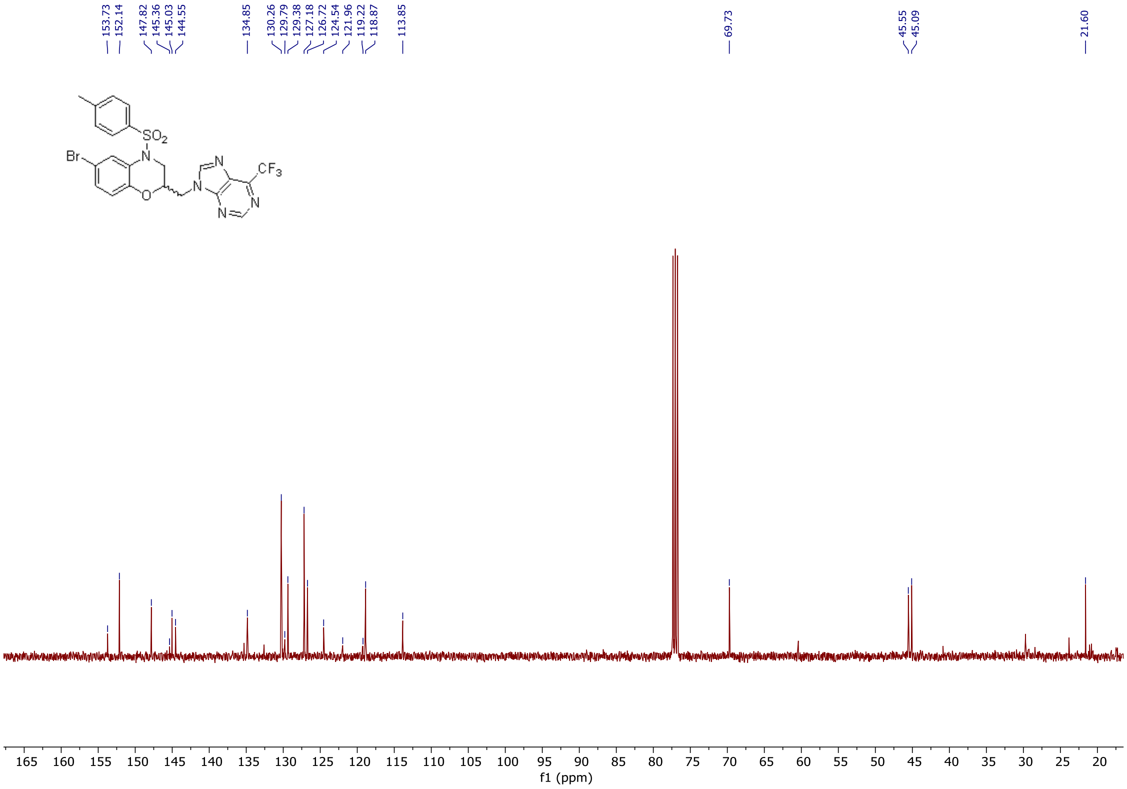

2-((6-Chloro-9*H*-purin-9-yl)methyl)-6-methyl-4-tosyl-3,4-dihydro-2*H*-benzo[b][1,4]oxazine (**11**).

^{1}H NMR

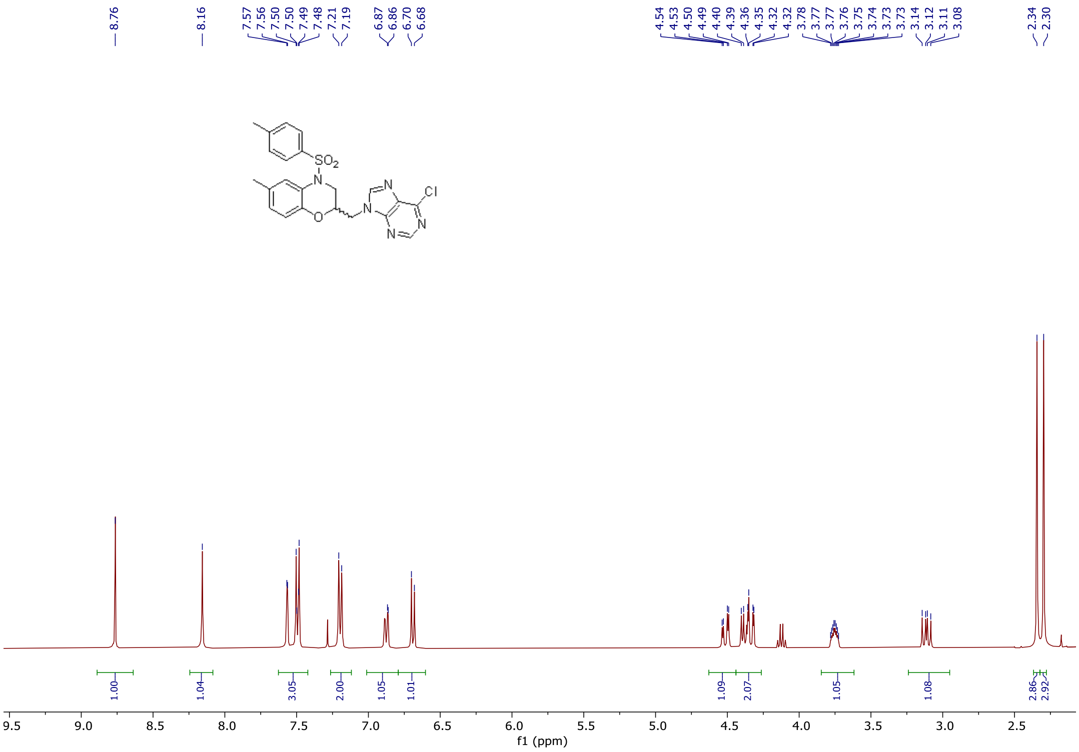

^{13}C NMR

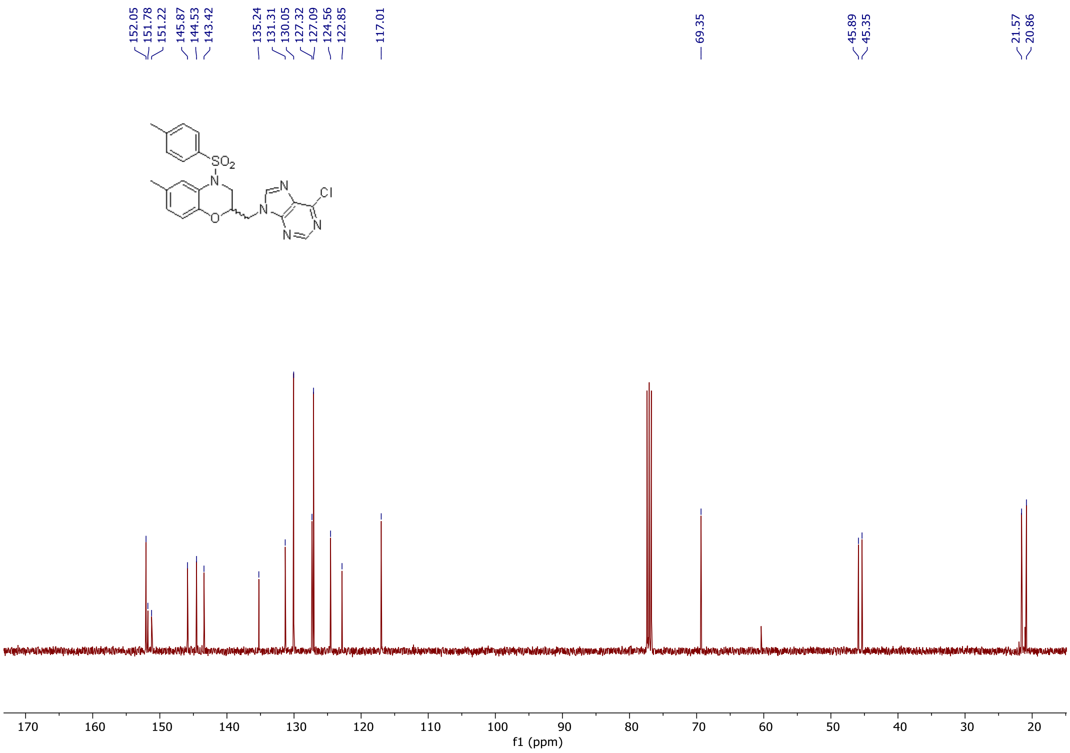

2-((2,6-Dichloro-9*H*-purin-9-yl)methyl)-6-methyl-4-tosyl-3,4-dihydro-2*H*-benzo[b][1,4]oxazine (**12**).

^{1}H NMR

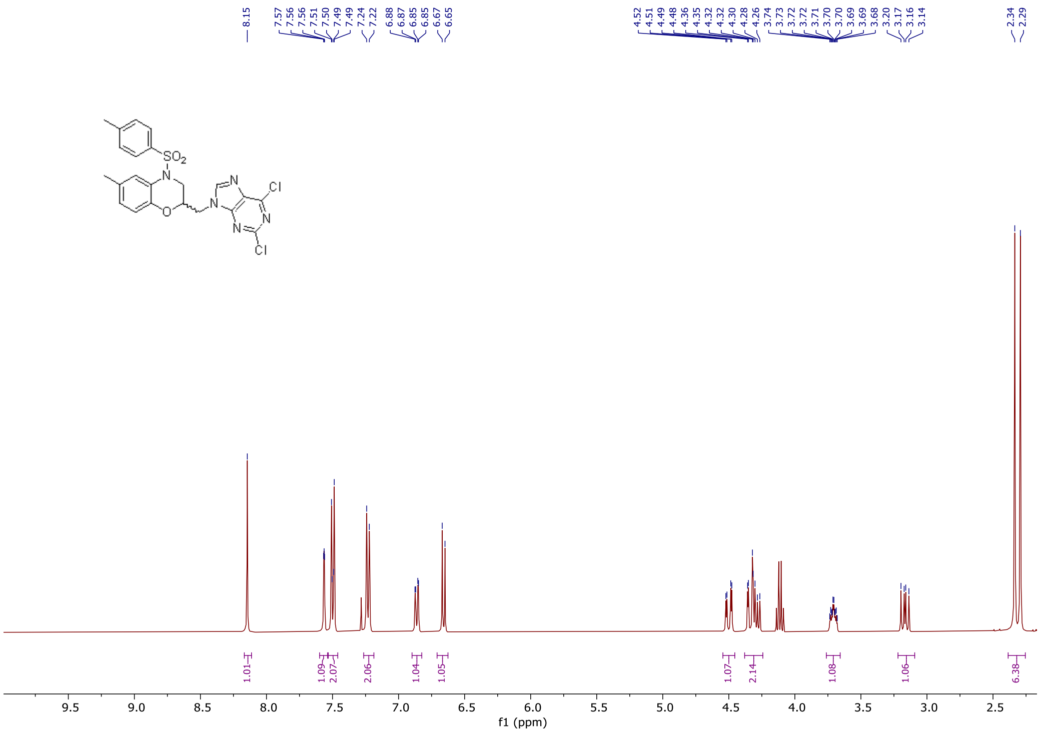

^{13}C NMR

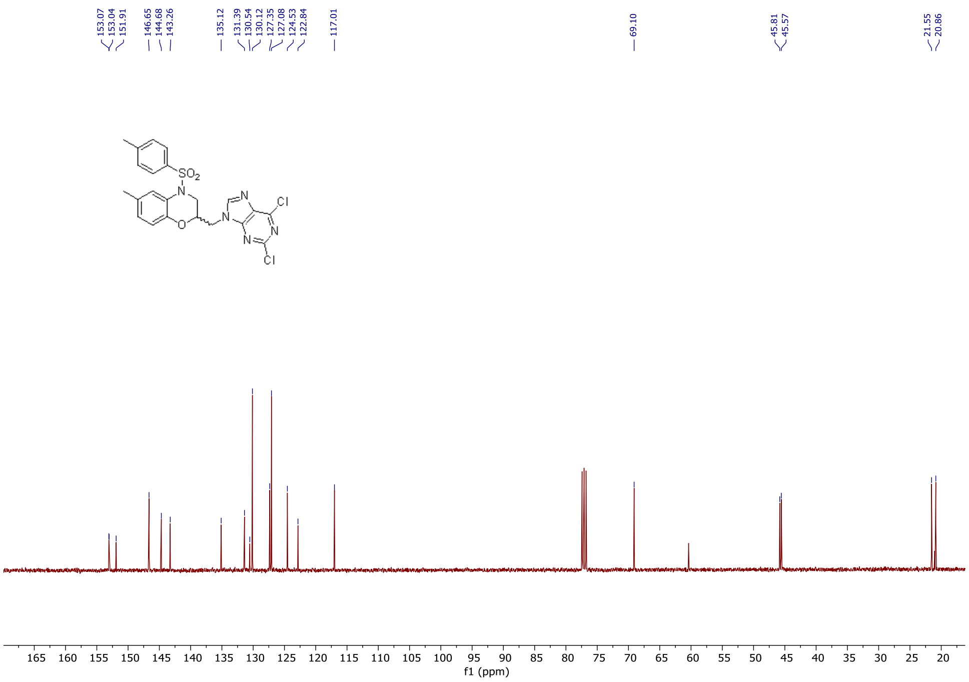

2-((6-Bromo-9*H*-purin-9-yl)methyl)-6-methyl-4-tosyl-3,4-dihydro-2*H*-benzo[b][1,4]oxazine (**13**).

^{1}H NMR

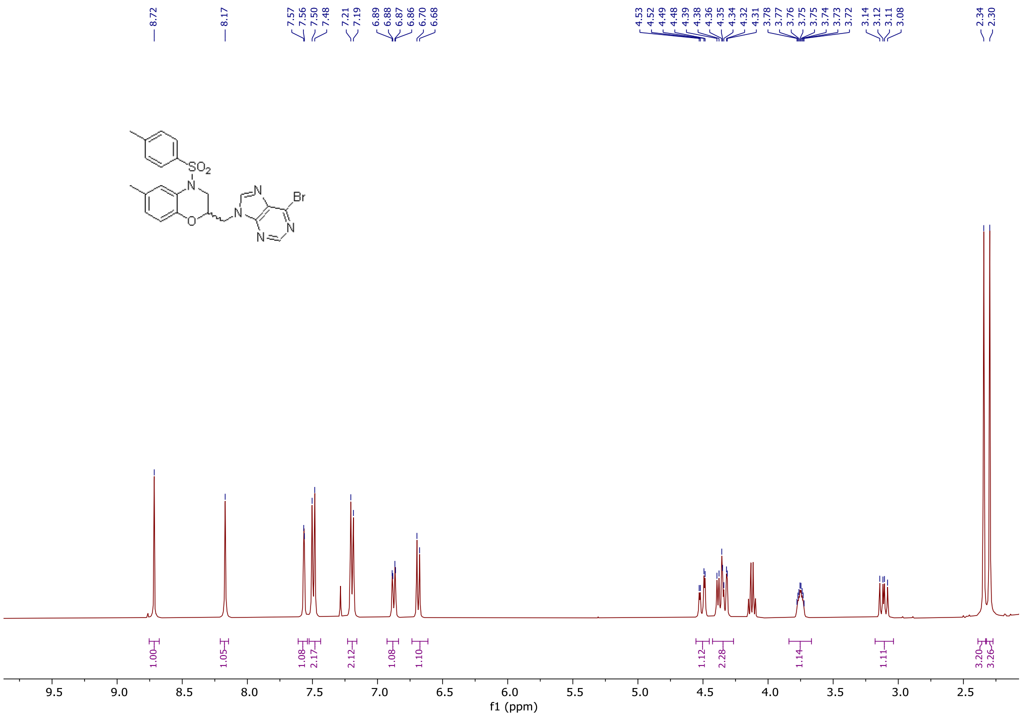

^{13}C NMR

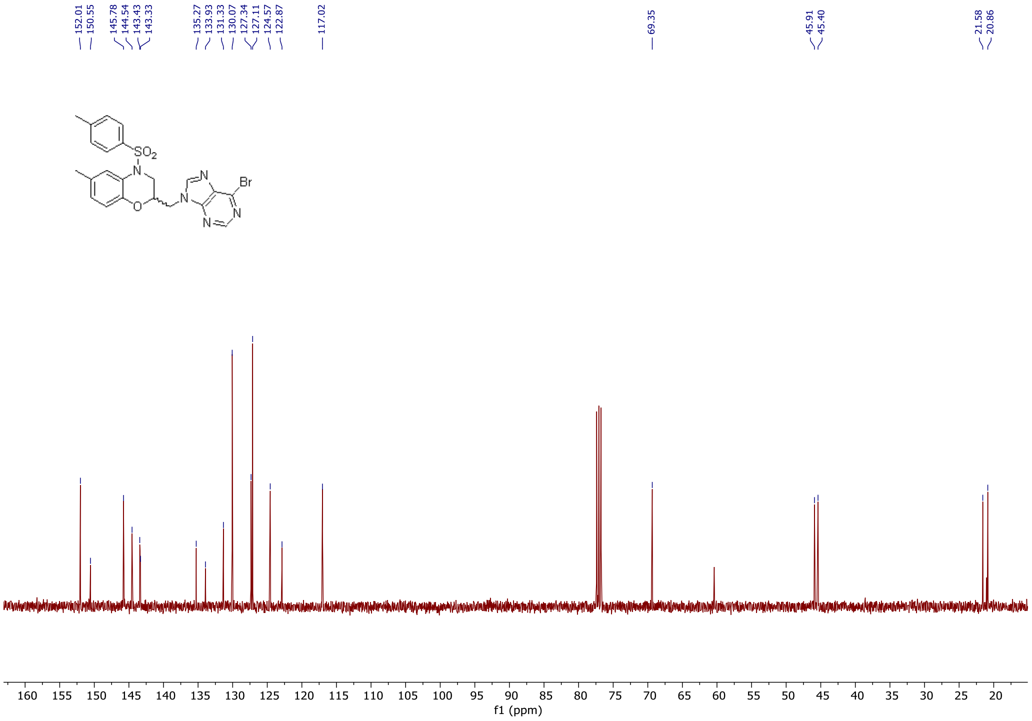

6-Methyl-4-tosyl-2-((6-(trifluoromethyl)-9*H*-purin-9-yl)methyl)-3,4-dihydro-2*H*-benzo[b][1,4]oxazine (**14**).

^{1}H NMR

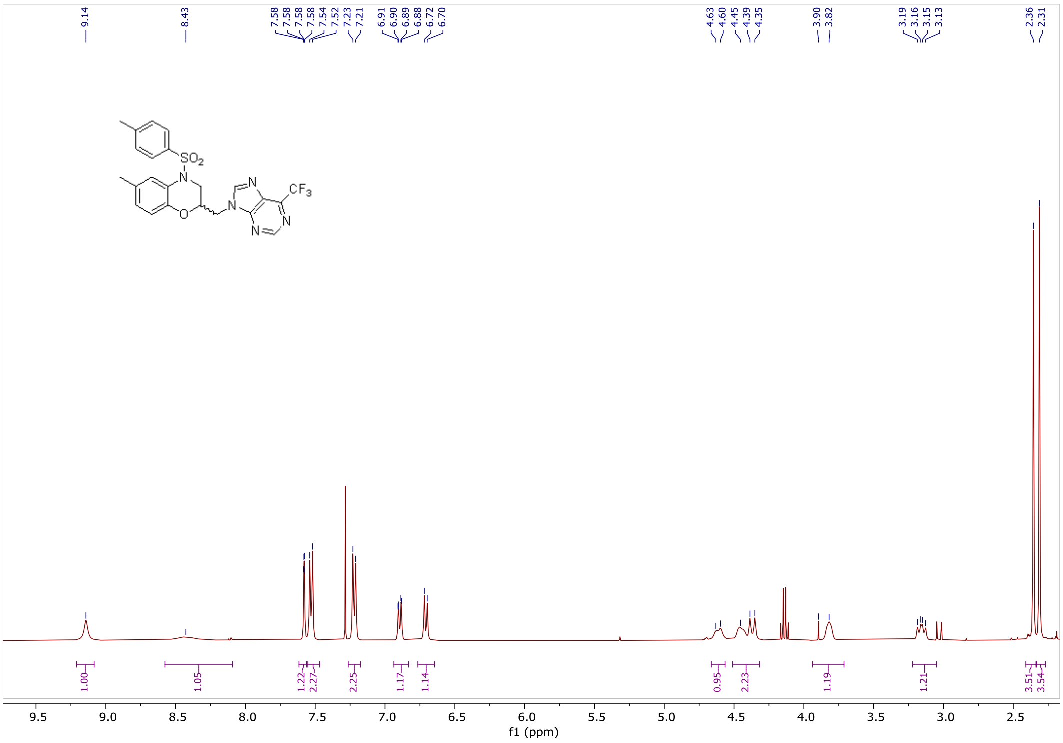

^{13}C NMR

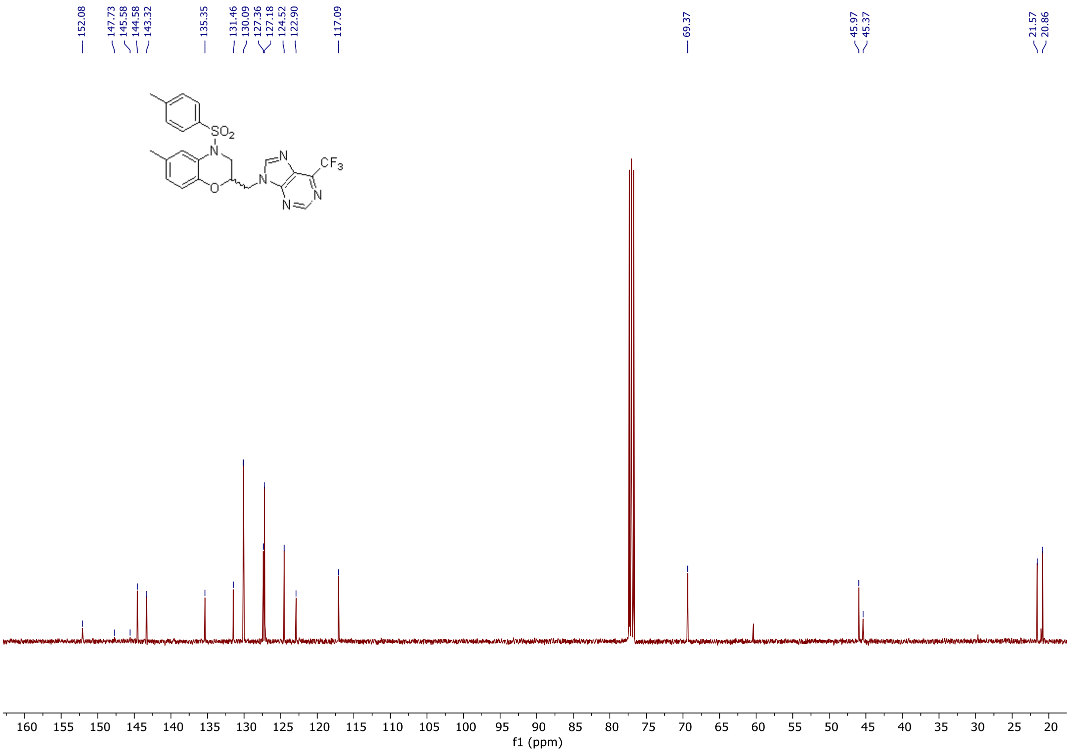

1.3. HRMS Spectra

(7-Chloro-3,4-dihydro-2*H*-benzo[b][1,4]oxazin-2-yl)methanol (**18**)

Elemental Composition Report **Page 1**

Single Mass Analysis
Tolerance = 10.0 PPM / DBE: min = -1.5, max = 50.0
Element prediction: Off
Number of isotope peaks used for i-FIT = 3

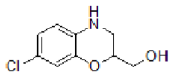

Monoisotopic Mass, Even Electron Ions
113 formula(e) evaluated with 1 results within limits (up to 50 best isotop
Elements Used:
C: 0-9 H: 0-1000 N: 0-3 O: 0-2 Na: 0-1 Cl: 0-2
CBS-101F1 11 (0.212) AM (Top,6, Ar,5000.0,0.00,1.00)
1: TOF MS ES+

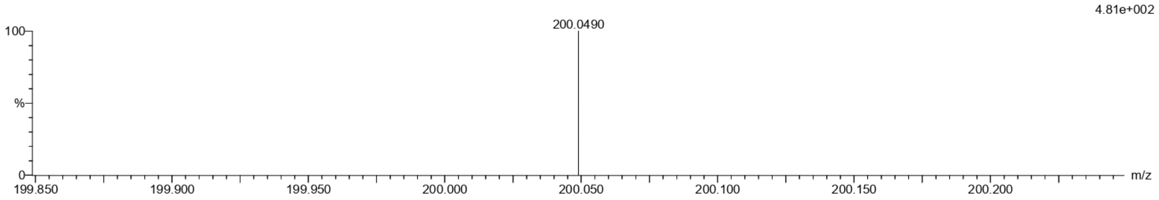

Minimum:				-1.5			
Maximum:		5.0	10.0	50.0			
Mass	Calc. Mass	mDa	PPM	DBE	i-FIT	i-FIT (Norm)	Formula
200.0490	200.0478	1.2	6.0	4.5	22.5	0.0	C9 H11 N O2 Cl

(6-Bromo-3,4-dihydro-2*H*-benzo[b][1,4]oxazin-2-yl)methanol (**19**)

Elemental Composition Report **Page 1**

Single Mass Analysis
Tolerance = 10.0 PPM / DBE: min = -1.5, max = 50.0
Element prediction: Off
Number of isotope peaks used for i-FIT = 3

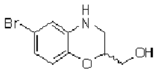

Monoisotopic Mass, Even Electron Ions
142 formula(e) evaluated with 1 results within limits (up to 50 best isotopic matches for each mass)
Elements Used:
C: 0-15 H: 0-1000 N: 0-3 O: 0-2 Na: 0-1 Br: 0-2

CBS-105 14 (0.283) AM (Cen,6, 50.00, Ar,5000.0,0.00,1.00)
1: TOF MS ES+

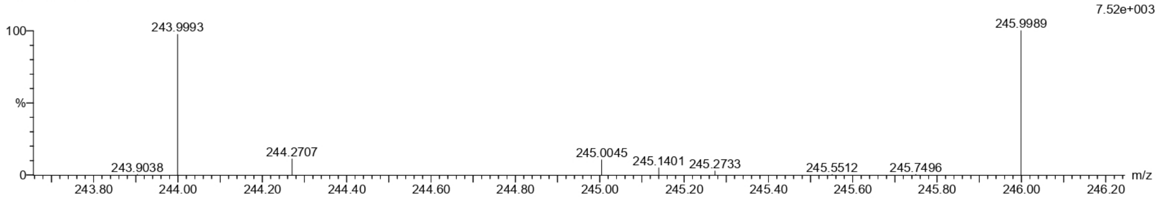

Minimum:				-1.5			
Maximum:		5.0	10.0	50.0			
Mass	Calc. Mass	mDa	PPM	DBE	i-FIT	i-FIT (Norm)	Formula
243.9993	243.9973	2.0	8.2	4.5	59.7	0.0	C9 H11 N O2 Br

(6-Methyl-3,4-dihydro-2*H*-benzo[b][1,4]oxazin-2-yl)methanol (**20**).

Elemental Composition Report **Page 1**

Single Mass Analysis
Tolerance = 10.0 PPM / DBE: min = -1.5, max = 50.0
Element prediction: Off
Number of isotope peaks used for i-FIT = 3

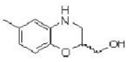

Monoisotopic Mass, Even Electron Ions
500 formula(e) evaluated with 3 results within limits (up to 50 best isotopic matches for each mass)
Elements Used:
C: 0-21 H: 0-1000 N: 0-5 O: 0-3 Na: 0-1 S: 0-1 Cl: 0-1 Br: 0-1

CBS-107 68 (1.462) AM (Top,1, Ar,5000.0,0.00,1.00)
1: TOF MS ES+

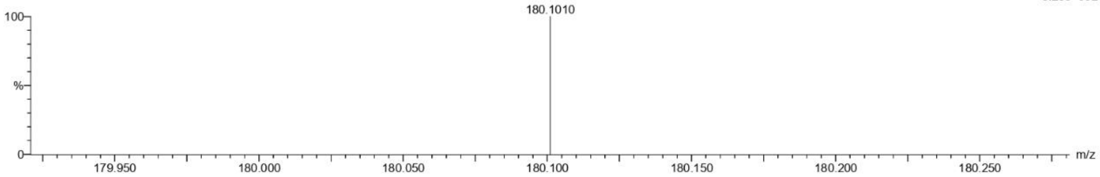

Minimum:				-1.5			
Maximum:		5.0	10.0	50.0			
Mass	Calc. Mass	mDa	PPM	DBE	i-FIT	i-FIT (Norm)	Formula
180.1010	180.1025	-1.5	-8.3	4.5	22.6	1.5	C10 H14 N O2
	180.1000	1.0	5.6	1.5	21.7	0.6	C8 H15 N O2 Na
	180.1016	-0.6	-3.3	0.5	22.6	1.5	C5 H15 N5 Cl

(7-Chloro-4-tosyl-3,4-dihydro-2*H*-benzo[b][1,4-]oxazin-2-yl)metanol (**21**).

Elemental Composition Report **Page 1**

Single Mass Analysis
Tolerance = 15.0 PPM / DBE: min = -50.0, max = 50.0
Element prediction: Off
Number of isotope peaks used for i-FIT = 3

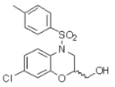

Monoisotopic Mass, Odd and Even Electron Ions
134 formula(e) evaluated with 1 results within limits (all results (up to 1000) for each mass)
Elements Used:
C: 0-16 H: 0-100 N: 0-1 O: 0-4 Na: 0-1 S: 0-1 Cl: 0-1
CBS-102 ACN FORMICO POS 877 (3.294) AM (Top,6, Ar,10000.0,0.00,0.00)
TOF MS ES+

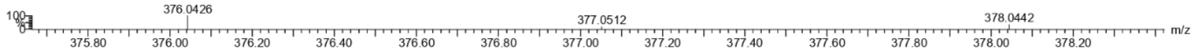

Minimum:				-50.0				
Maximum:		40.0	15.0	50.0				
Mass	Calc. Mass	mDa	PPM	DBE	i-FIT	Norm	Conf(%)	Formula
376.0426	376.0386	4.0	10.6	8.5	20.4	n/a	n/a	C16 H16 N O4 Na S Cl

(6-Bromo-4-tosyl-3,4-dihydro-2*H*-benzo[b][1,4-]oxazin-2-yl)methanol (**22**).

Elemental Composition Report **Page 1**

Single Mass Analysis
Tolerance = 10.0 PPM / DBE: min = -1.5, max = 50.0
Element prediction: Off
Number of isotope peaks used for i-FIT = 3

Monoisotopic Mass, Even Electron Ions
286 formula(e) evaluated with 2 results within limits (up to 50 best isotopic matches for each mass)
Elements Used:
C: 0-16 H: 0-1000 N: 0-1 O: 0-4 Na: 0-1 S: 0-1 Cl: 0-2 Br: 0-1

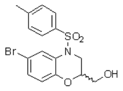

CBS-108neg 22 (0.498) AM (Top,1, Ar,5000.0,0.00,1.00)
1: TOF MS ES-

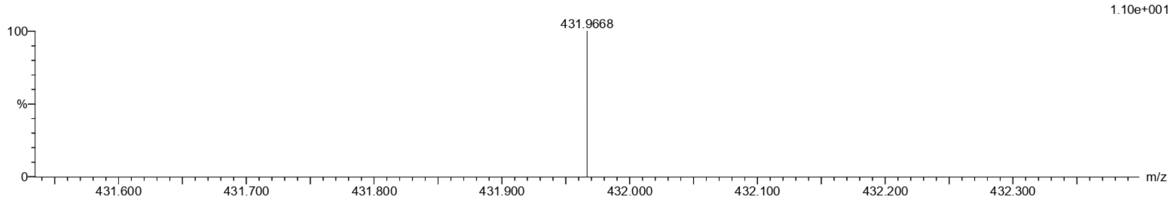

Minimum:				-1.5			
Maximum:		5.0	10.0	50.0			
Mass	Calc. Mass	mDa	PPM	DBE	i-FIT	i-FIT (Norm)	Formula
431.9668	431.9672	-0.4	-0.9	8.5	15.0	0.7	C16 H16 N O4 S Cl Br
	431.9648	2.0	4.6	5.5	15.1	0.7	C14 H17 N O4 Na S Cl Br

(6-Methyl-4-tosyl-3,4-dihydro-2*H*-benzo[b][1,4-]oxazin-2-yl)methanol (**23**)

Elemental Composition Report **Page 1**

Single Mass Analysis
Tolerance = 10.0 PPM / DBE: min = -1.5, max = 50.0
Element prediction: Off
Number of isotope peaks used for i-FIT = 3

Monoisotopic Mass, Even Electron Ions
135 formula(e) evaluated with 1 results within limits (up to 50 best isotopic matches for each mass)
Elements Used:
C: 0-17 H: 0-1000 N: 0-4 O: 0-4 Na: 0-1 S: 0-1

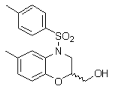

CBS-109 17 (0.334) AM (Top,6, Ar,5000.0,0.00,1.00); Cm (16:20)
1: TOF MS ES+

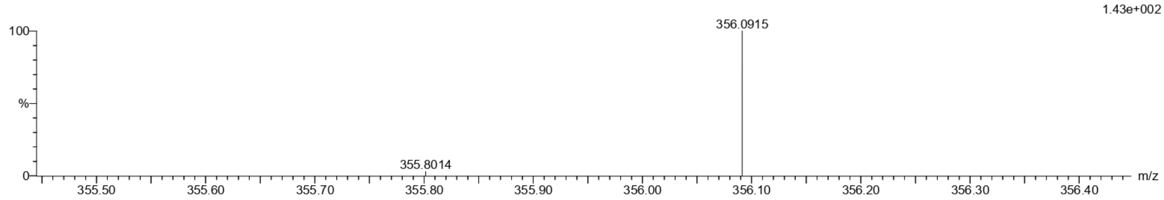

Minimum:				-1.5			
Maximum:		5.0	10.0	50.0			
Mass	Calc. Mass	mDa	PPM	DBE	i-FIT	i-FIT (Norm)	Formula
356.0915	356.0932	-1.7	-4.8	8.5	19.5	0.0	C17 H19 N O4 Na S

7-Chloro-2-((6-chloro-9*H*-purin-9-yl)methyl)-4-tosyl-3,4-dihydro-2*H*-benzo[b][1,4]oxazine (**3**).

Elemental Composition Report **Page 1**

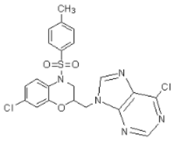

Single Mass Analysis
Tolerance = 15.0 PPM / DBE: min = -50.0, max = 50.0
Element prediction: Off
Number of isotope peaks used for i-FIT = 3

Monoisotopic Mass, Even Electron Ions
633 formula(e) evaluated with 2 results within limits (all results (up to 1000) for each mass)
Elements Used:
C: 0-21 H: 0-100 N: 0-5 O: 0-4 Na: 0-1 S: 0-1 Cl: 0-2
CBS-103-2 22 (0.099) AM (Top,6, Ar,10000.0,0.00,0.00)
TOF MS ES+

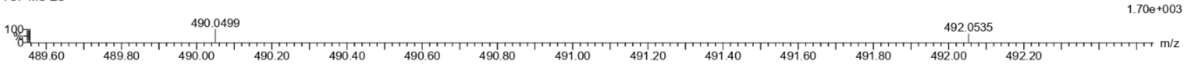

Mass	Calc. Mass	mDa	PPM	DBE	i-FIT	Norm	Conf(%)	Formula
Minimum:				-50.0				
Maximum:		40.0	15.0	50.0				
490.0499	490.0507	-0.8	-1.6	14.5	29.0	0.688	50.28	C21 H18 N5 O3 S Cl2
	490.0483	1.6	3.3	11.5	29.0	0.699	49.72	C19 H19 N5 O3 Na S Cl2

7-Chloro-2-((2,6-dichloro-9*H*-purin-9-yl)methyl)-4-tosyl-3,4-dihydro-2*H*-benzo[b][1,4]oxazine (**4**)

Elemental Composition Report **Page 1**

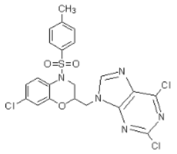

Single Mass Analysis
Tolerance = 15.0 PPM / DBE: min = -50.0, max = 50.0
Element prediction: Off
Number of isotope peaks used for i-FIT = 3

Monoisotopic Mass, Even Electron Ions
742 formula(e) evaluated with 2 results within limits (all results (up to 1000) for each mass)
Elements Used:
C: 0-21 H: 0-100 N: 0-5 O: 0-4 Na: 0-1 S: 0-1 Cl: 0-3
CBS-104 407 (1.538) AM (Top,1, Ar,10000.0,556.27,0.00)
TOF MS ES+

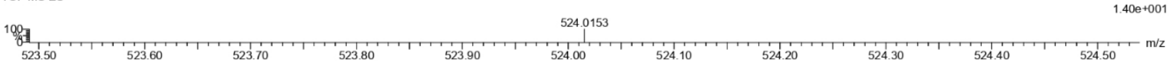

Mass	Calc. Mass	mDa	PPM	DBE	i-FIT	Norm	Conf(%)	Formula
Minimum:				-50.0				
Maximum:		40.0	15.0	50.0				
524.0153	524.0118	3.5	6.7	14.5	15.0	0.535	58.54	C21 H17 N5 O3 S Cl3
	524.0094	5.9	11.3	11.5	15.3	0.880	41.46	C19 H18 N5 O3 Na S Cl3

7-Chloro-2-((6-bromo-9*H*-purin-9-yl)methyl)-4-tosyl-3,4-dihydro-2*H*-benzo[b][1,4]oxazine (**5**).

Elemental Composition Report **Page 1**

Single Mass Analysis
Tolerance = 10.0 PPM / DBE: min = -1.5, max = 50.0
Element prediction: Off
Number of isotope peaks used for i-FIT = 3

Monoisotopic Mass, Even Electron Ions
410 formula(e) evaluated with 2 results within limits (up to 50 best isotopic matches for each mass)
Elements Used:
C: 0-21 H: 0-1000 N: 0-5 O: 0-3 Na: 0-1 S: 0-1 Cl: 0-1 Br: 0-1

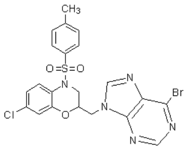

CBS-106 11 (0.212) AM (Top,1, Ar,5000.0,0.00,1.00)
1: TOF MS ES+

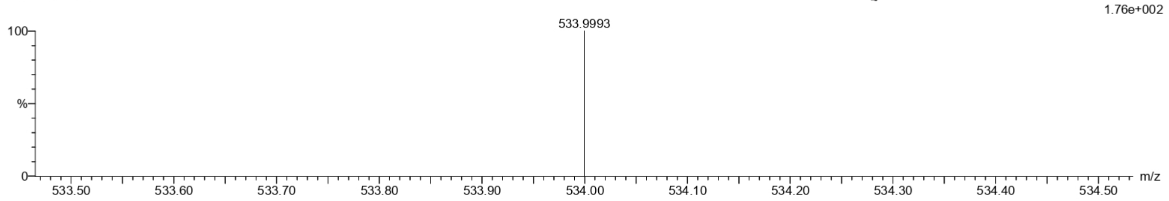

Minimum:				-1.5			
Maximum:		5.0	10.0	50.0			
Mass	Calc. Mass	mDa	PPM	DBE	i-FIT	i-FIT (Norm)	Formula
533.9993	534.0002	-0.9	-1.7	14.5	21.4	0.7	C21 H18 N5 O3 S Cl Br
	533.9978	1.5	2.8	11.5	21.5	0.7	C19 H19 N5 O3 Na S Cl Br

7-Chloro-4-tosyl-2-((6-(trifluoromethyl)-9*H*-purin-9-yl)methyl)-3,4-dihydro-2*H*-benzo[b][1,4]oxazine (**6**).

Elemental Composition Report **Page 1**

Single Mass Analysis
Tolerance = 10.0 PPM / DBE: min = -1.5, max = 50.0
Element prediction: Off
Number of isotope peaks used for i-FIT = 3

Monoisotopic Mass, Even Electron Ions
1524 formula(e) evaluated with 2 results within limits (up to 50 best isotopic matches for each mass)
Elements Used:
C: 0-22 H: 0-1000 N: 0-5 O: 0-4 Na: 0-1 S: 0-1 Cl: 0-2 F: 0-3

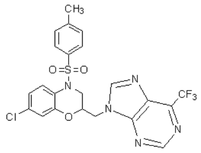

CBS-110 neg 12 (0.267) AM (Cen,6, 90.00, Ar,5000.0,0.00,1.00); Cm (9:16)
1: TOF MS ES-

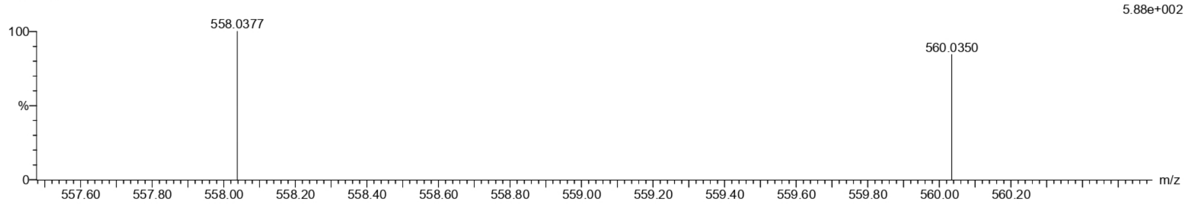

Minimum:				-1.5			
Maximum:		5.0	10.0	50.0			
Mass	Calc. Mass	mDa	PPM	DBE	i-FIT	i-FIT (Norm)	Formula
558.0377	558.0381	-0.4	-0.7	14.5	21.6	0.5	C22 H17 N5 O3 S Cl2 F3
	558.0357	2.0	3.6	11.5	21.9	0.9	C20 H18 N5 O3 Na S Cl2 F3

6-Bromo-2-((6-chloro-9*H*-purin-9-yl)methyl)-4-tosyl-3,4-dihydro-2*H*-benzo[b][1,4]oxazine (**7**)

Elemental Composition Report **Page 1**

Single Mass Analysis
Tolerance = 10.0 PPM / DBE: min = -1.5, max = 50.0
Element prediction: Off
Number of isotope peaks used for i-FIT = 3

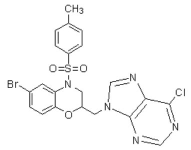

Monoisotopic Mass, Even Electron Ions
410 formula(e) evaluated with 2 results within limits (up to 50 best isotopic matches for each mass)
Elements Used:
C: 0-21 H: 0-1000 N: 0-5 O: 0-3 Na: 0-1 S: 0-1 Cl: 0-1 Br: 0-1

CBS-111 15 (0.300) AM (Top,1, Ar,5000.0,0.00,1.00)
1: TOF MS ES+

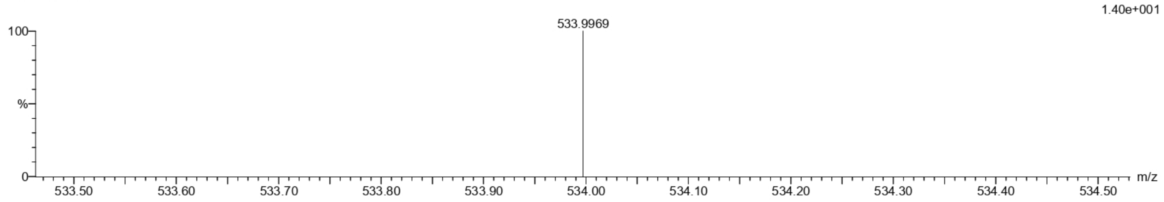

Minimum:				-1.5			
Maximum:		5.0	10.0	50.0			

Mass	Calc. Mass	mDa	PPM	DBE	i-FIT	i-FIT (Norm)	Formula
533.9969	534.0002	-3.3	-6.2	14.5	16.0	0.7	C21 H18 N5 O3 S Cl Br
	533.9978	-0.9	-1.7	11.5	15.9	0.7	C19 H19 N5 O3 Na S Cl Br

6-Bromo-2-((2,6-dichloro-9*H*-purin-9-yl)methyl)-4-tosyl-3,4-dihydro-2*H*-benzo[b][1,4]oxazine (**8**)

Elemental Composition Report **Page 1**

Single Mass Analysis
Tolerance = 10.0 PPM / DBE: min = -1.5, max = 50.0
Element prediction: Off
Number of isotope peaks used for i-FIT = 3

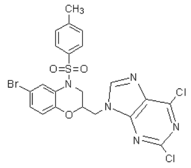

Monoisotopic Mass, Even Electron Ions
602 formula(e) evaluated with 2 results within limits (up to 50 best isotopic matches for each mass)
Elements Used:
C: 0-21 H: 0-1000 N: 0-5 O: 0-3 Na: 0-1 S: 0-1 Cl: 0-2 Br: 0-1

CBS-112 7 (0.124) AM (Top,1, Ar,5000.0,0.00,1.00); Cm (6:11)
1: TOF MS ES+

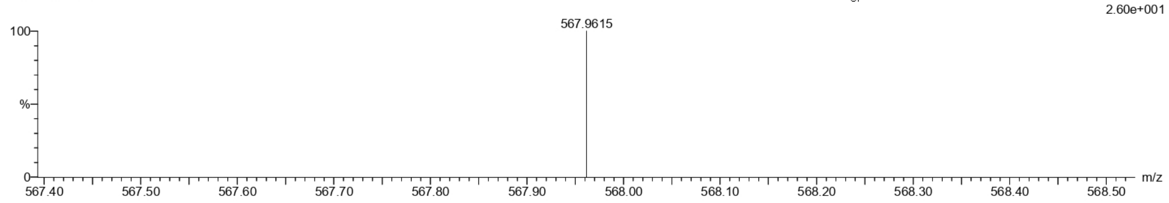

Minimum:				-1.5			
Maximum:		5.0	10.0	50.0			

Mass	Calc. Mass	mDa	PPM	DBE	i-FIT	i-FIT (Norm)	Formula
567.9615	567.9613	0.2	0.4	14.5	17.4	0.7	C21 H17 N5 O3 S Cl2 Br
	567.9588	2.7	4.8	11.5	17.5	0.7	C19 H18 N5 O3 Na S Cl2 Br

6-Bromo-2-((6-bromo-9*H*-purin-9-yl)methyl)-4-tosyl-3,4-dihydro-2*H*-benzo[b][1,4]oxazine (**9**)

Elemental Composition Report **Page 1**

Single Mass Analysis
Tolerance = 10.0 PPM / DBE: min = -1.5, max = 50.0
Element prediction: Off
Number of isotope peaks used for i-FIT = 3

Monoisotopic Mass, Even Electron Ions
506 formula(e) evaluated with 2 results within limits (up to 50 best isotopic matches for each mass)
Elements Used:
C: 0-21 H: 0-1000 N: 0-6 O: 0-4 Na: 0-1 S: 0-1 Br: 0-2

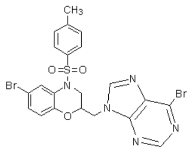

CBS-116 2 (0.053) AM (Top,1, Ar,5000.0,0.00,1.00)
1: TOF MS ES+

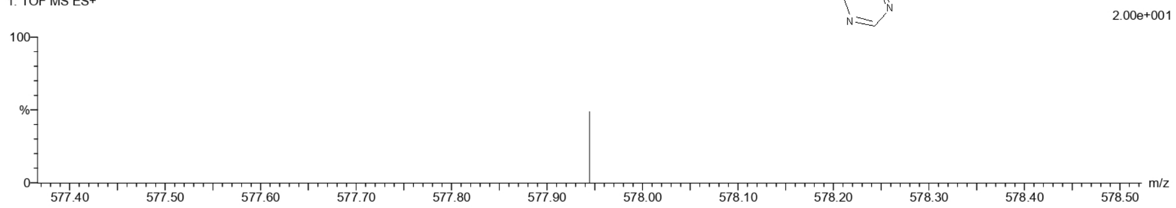

Mass	Calc. Mass	mDa	PPM	DBE	i-FIT	i-FIT (Norm)	Formula
Minimum:				-1.5			
Maximum:		5.0	10.0	50.0			
577.9446	577.9497	-5.1	-8.8	14.5	17.2	0.8	C21 H18 N5 O3 S Br2
	577.9473	-2.7	-4.7	11.5	17.0	0.6	C19 H19 N5 O3 Na S Br2

6-Bromo-4-tosyl-2-((6-(trifluoromethyl)-9*H*-purin-9-yl)methyl)-3,4-dihydro-2*H*-benzo[b][1,4]oxazine (**10**)

Elemental Composition Report **Page 1**

Single Mass Analysis
Tolerance = 10.0 PPM / DBE: min = -1.5, max = 50.0
Element prediction: Off
Number of isotope peaks used for i-FIT = 3

Monoisotopic Mass, Even Electron Ions
217 formula(e) evaluated with 2 results within limits (up to 50 best isotopic matches for each mass)
Elements Used:
C: 0-22 H: 0-1000 N: 0-5 O: 0-3 F: 3-3 Na: 0-1 S: 0-1 Br: 0-1

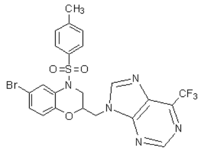

CBS118 6 (0.107) AM (Top,1, Ar,5000.0,0.00,1.00)
1: TOF MS ES+

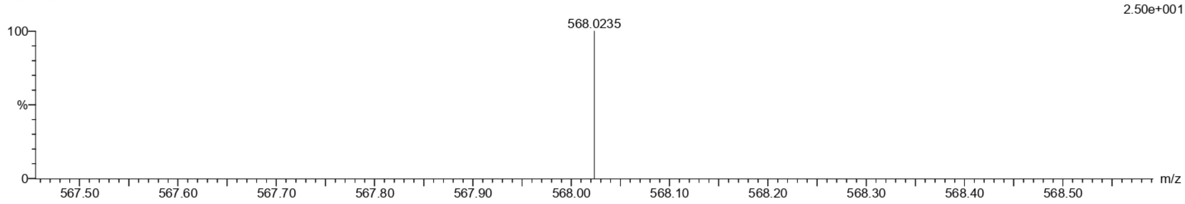

Mass	Calc. Mass	mDa	PPM	DBE	i-FIT	i-FIT (Norm)	Formula
Minimum:				-1.5			
Maximum:		5.0	10.0	50.0			
568.0235	568.0266	-3.1	-5.5	14.5	17.1	0.7	C22 H18 N5 O3 F3 S Br
	568.0242	-0.7	-1.2	11.5	17.0	0.7	C20 H19 N5 O3 F3 Na S Br

2-((6-Chloro-9*H*-purin-9-yl)methyl)-6-methyl-4-tosyl-3,4-dihydro-2*H*-benzo[b][1,4]oxazine (**11**).

Elemental Composition Report **Page 1**

Single Mass Analysis
Tolerance = 10.0 PPM / DBE: min = -1.5, max = 50.0
Element prediction: Off
Number of isotope peaks used for i-FIT = 3

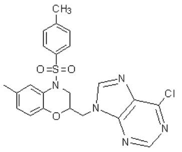

Monoisotopic Mass, Even Electron Ions
218 formula(e) evaluated with 2 results within limits (up to 50 best isotopic matches for each mass)
Elements Used:
C: 0-22 H: 0-1000 N: 0-5 O: 0-3 Na: 0-1 S: 0-1 Cl: 0-1
CBS-115 82 (1.782) AM (Top,1, Ar,5000.0,0.00,1.00)
1: TOF MS ES+

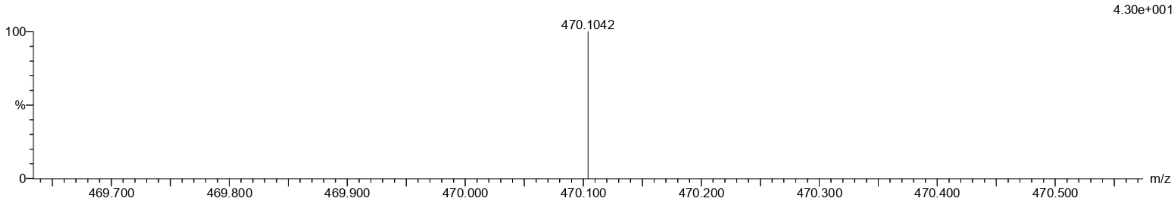

Minimum:				-1.5			
Maximum:		5.0	10.0	50.0			
Mass	Calc. Mass	mDa	PPM	DBE	i-FIT	i-FIT (Norm)	Formula
470.1042	470.1054	-1.2	-2.6	14.5	17.3	0.7	C22 H21 N5 O3 S Cl
	470.1030	1.2	2.6	11.5	17.2	0.7	C20 H22 N5 O3 Na S Cl

2-((2,6-Dichloro-9*H*-purin-9-yl)methyl)-6-methyl-4-tosyl-3,4-dihydro-2*H*-benzo[b][1,4]oxazine (**12**).

Elemental Composition Report **Page 1**

Single Mass Analysis
Tolerance = 10.0 PPM / DBE: min = -1.5, max = 50.0
Element prediction: Off
Number of isotope peaks used for i-FIT = 3

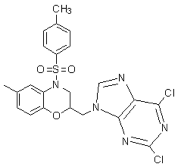

Monoisotopic Mass, Even Electron Ions
527 formula(e) evaluated with 2 results within limits (up to 50 best isotopic matches for each mass)
Elements Used:
C: 0-22 H: 0-1000 N: 0-6 O: 0-4 Na: 0-1 S: 0-1 Cl: 0-2
CBS-117 10 (0.195) AM (Top,1, Ar,5000.0,0.00,1.00)
1: TOF MS ES+

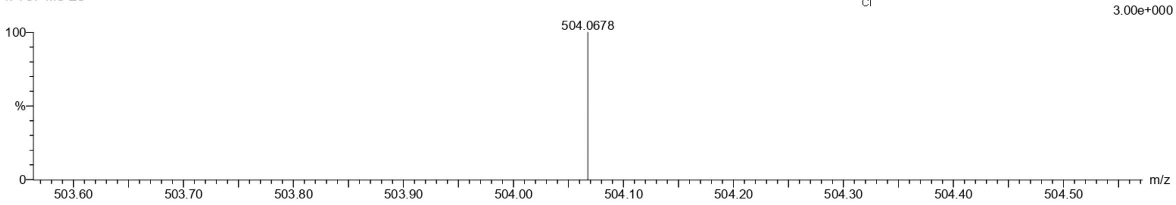

Minimum:				-1.5			
Maximum:		5.0	10.0	50.0			
Mass	Calc. Mass	mDa	PPM	DBE	i-FIT	i-FIT (Norm)	Formula
504.0678	504.0664	1.4	2.8	14.5	12.9	0.7	C22 H20 N5 O3 S Cl2
	504.0640	3.8	7.5	11.5	13.0	0.7	C20 H21 N5 O3 Na S Cl2

2-((6-Bromo-9*H*-purin-9-yl)methyl)-6-methyl-4-tosyl-3,4-dihydro-2*H*-benzo[b][1,4]oxazine (**13**).

Elemental Composition Report **Page 1**

Single Mass Analysis
Tolerance = 10.0 PPM / DBE: min = -1.5, max = 50.0
Element prediction: Off
Number of isotope peaks used for i-FIT = 3

Monoisotopic Mass, Even Electron Ions
217 formula(e) evaluated with 2 results within limits (up to 50 best isotopic matches for each mass)
Elements Used:
C: 0-22 H: 0-1000 N: 0-5 O: 0-3 Na: 0-1 S: 0-1 Br: 0-1

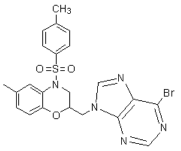

CBS-119 3 (0.053) AM (Top,1, Ar,5000.0,0.00,1.00)
1: TOF MS ES+

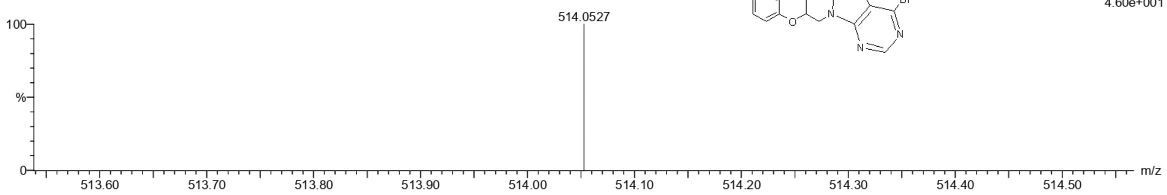

Minimum:				-1.5			
Maximum:		5.0	10.0	50.0			
Mass	Calc. Mass	mDa	PPM	DBE	i-FIT	i-FIT (Norm)	Formula
514.0527	514.0548	-2.1	-4.1	14.5	18.3	0.7	C22 H21 N5 O3 S Br
	514.0524	0.3	0.6	11.5	18.2	0.6	C20 H22 N5 O3 Na S Br

6-Methyl-4-tosyl-2-((6-(trifluoromethyl)-9*H*-purin-9-yl)methyl)-3,4-dihydro-2*H*-benzo[b][1,4]oxazine (**14**).

Elemental Composition Report **Page 1**

Single Mass Analysis
Tolerance = 10.0 PPM / DBE: min = -1.5, max = 50.0
Element prediction: Off
Number of isotope peaks used for i-FIT = 3

Monoisotopic Mass, Even Electron Ions
121 formula(e) evaluated with 2 results within limits (up to 50 best isotopic matches for each mass)
Elements Used:
C: 0-23 H: 0-1000 N: 0-5 O: 0-3 F: 3-3 Na: 0-1 S: 0-1
CBS-120 22 (0.460) AM (Top,1, Ar,5000.0,0.00,1.00)
1: TOF MS ES+

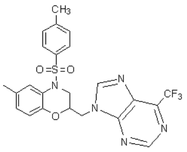

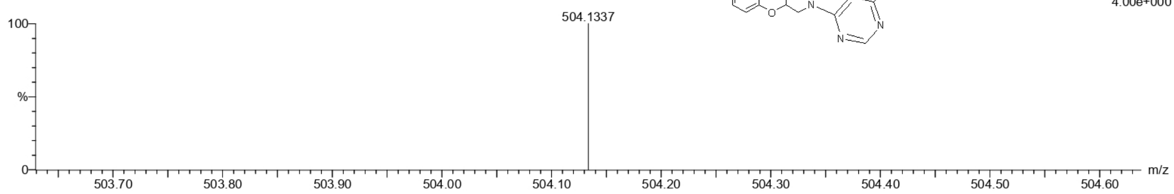

Minimum:				-1.5			
Maximum:		5.0	10.0	50.0			
Mass	Calc. Mass	mDa	PPM	DBE	i-FIT	i-FIT (Norm)	Formula
504.1337	504.1317	2.0	4.0	14.5	12.7	0.7	C23 H21 N5 O3 F3 S
	504.1293	4.4	8.7	11.5	12.8	0.7	C21 H22 N5 O3 F3 Na S

2. Biology

2.1. IC_{50} Curves for compounds 3-14

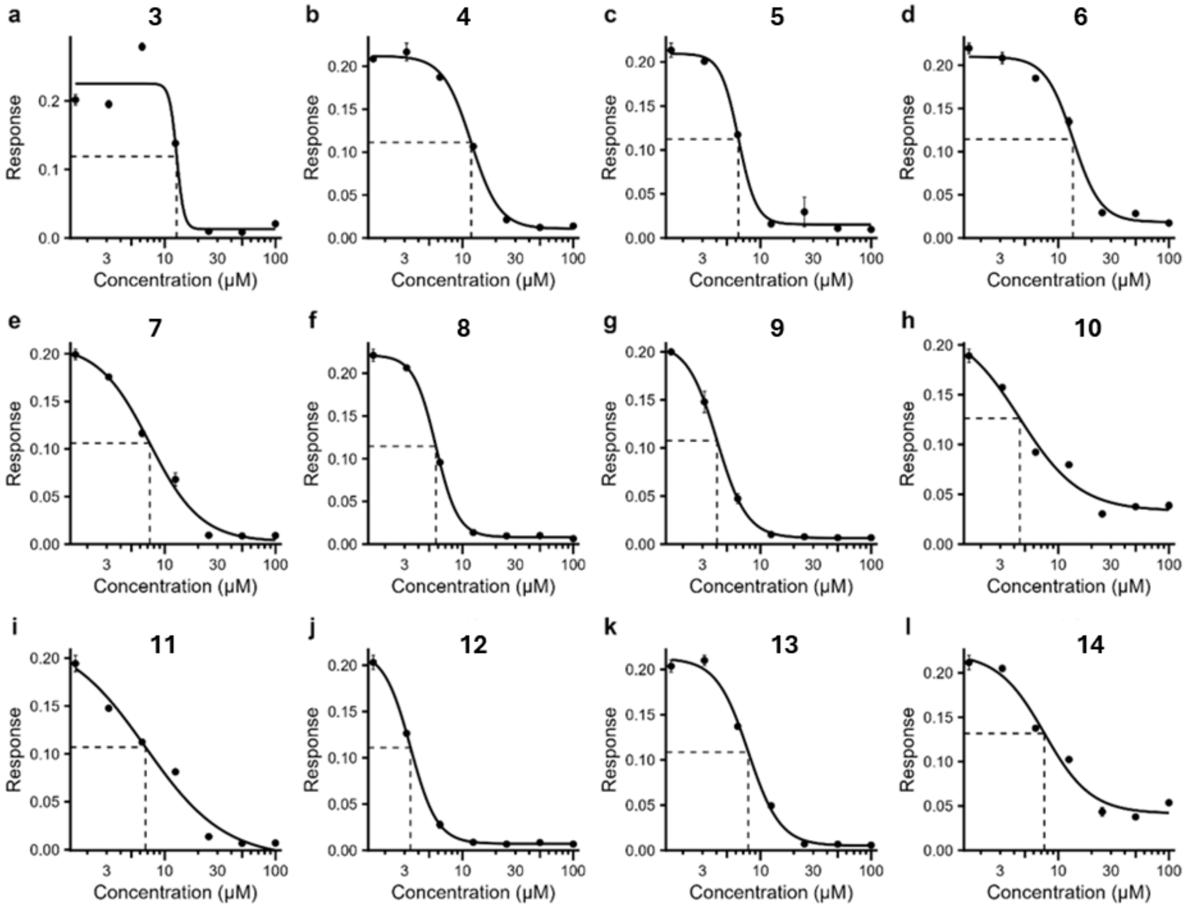

Figure S1. Dose–response curves showing IC_{50} determination of compounds **3**-**14** evaluated in MCF-7 cells. Each data point represents the mean response ± SD from three independent replicates (n = 3). Curves were generated using a four-parameter log-logistic model. Dashed lines indicate the IC_{50} value for each compound.

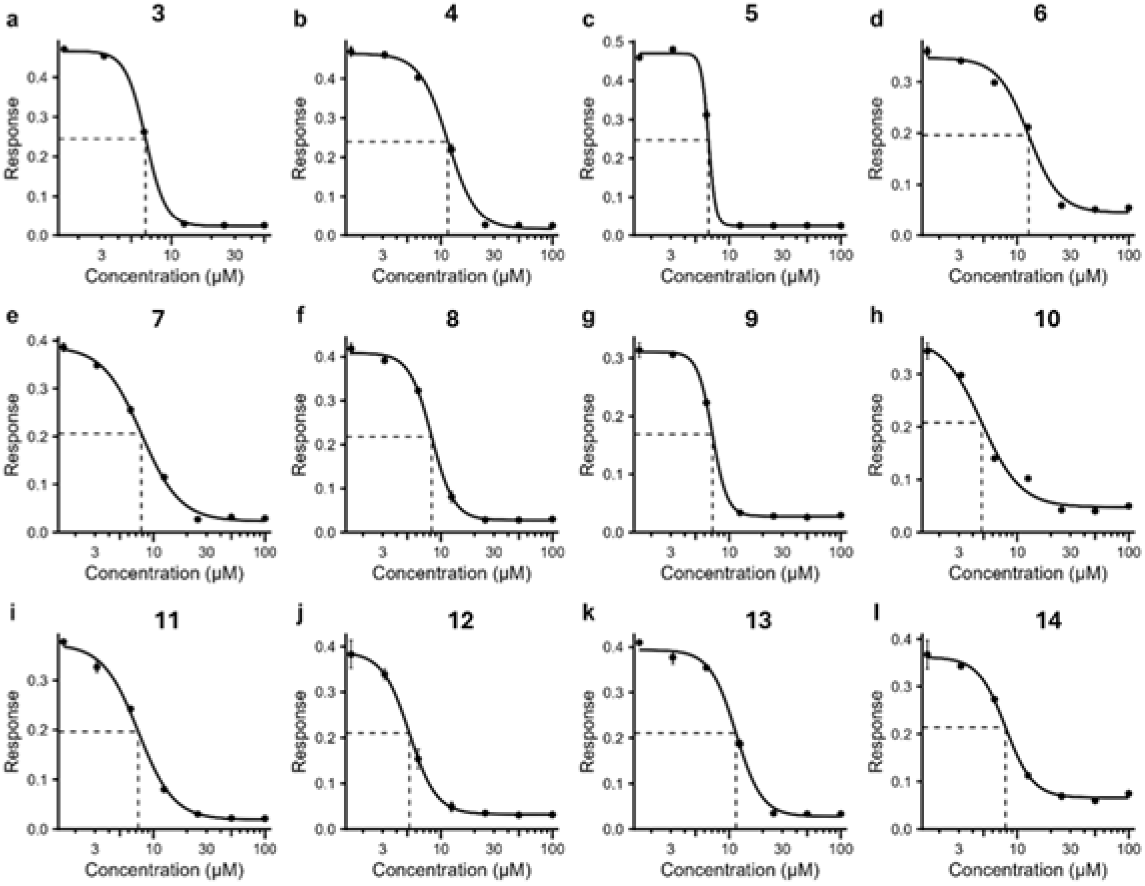

Figure S2. Dose–response curves showing IC_{50} determination of compounds **3-14** evaluated in HCT116 cells. Each data point represents the mean response ± SD from three independent replicates (*n* = 3). Curves were generated using a four-parameter log-logistic model. Dashed lines indicate the IC_{50} value for each compound.

2.2. Kinase inhibition assay

The compounds were dissolved to 5×10^{-03} M in volumes of 100% DMSO. Aliquots were further diluted to 5×10^{-03} M/100% DMSO. 100 µl of each of the (diluted) stock solutions were transferred in a 96 well master plate. Prior to the testing, 3 x 10 µl from each well of the master plate were aliquoted with a 96 channel pipettor into three identical copy plates. For each test run a separate copy plate was used.

In the process, 90 µl H_2O were added to each well of the copy plate. To minimize potential compound precipitation, the H_2O was added to each well only a few minutes before the transfer of the compound solutions into the assay plates. The plate was shaken thoroughly, resulting in a compound dilution plate with a compound concentration of 5×10^{-4} M/10 %

DMSO and 5 x 10^{-5} M/10 % DMSO. This plate was used for the transfer of 5 µl compound solution into the assay plates. The final volume of the assay was 50 µl. All compounds were tested at 5 x 10^{-6} M and 5 x 10^{-5} M in singlicate in every kinase assay. The final DMSO concentration in the reaction cocktails was 1 % in all cases.

Recombinant Protein Kinases

All protein kinases provided by RBE were expressed in Sf9 insect cells or in *E. coli* as recombinant GST-fusion proteins or His-tagged proteins, either as full-length or enzymatically active fragments. All kinases were produced from human cDNAs and purified by either GSH-affinity chromatography or immobilized metal affinity chromatography. The purity of the protein kinases was examined by SDS-PAGE/Coomassie staining, the identity was checked by mass spectroscopy.

Kinases from external vendors (CAR = Carna Biosciences Inc.; INV = Life Technologies (Invitrogen Corporation); MIL = Merck-Millipore (Millipore Corporation), (see Table S1) were expressed, purified and quality-controlled by virtue of the vendors readings.

Table S1. Assay parameters for the tested protein kinases.

Kinase Name	*Kinase PQ Lot*	*Kinase External Vendor Lot*	*Kinase Conc. (ng/50µl)*	*Kinase Conc. (nM) **	*ATP Conc. (µM)*	*Substrate Name*	*Subs. Lot*	*Subs. Conc (µg/50µl)*
AMPKalpha1	001,9		200	43,4	3,0	RBER-CHKtide	120	2,0
HER2	012,10		50	10,6	1,0	Poly(Glu, Tyr)4:1	SIG_20K5903	0,125
ERK2	008,8		10	4,8	0,3	RBER-CHKtide	120	2,0
JNK1	005,16		5	2,3	0,3	ATF2	14	0,25
LKB1/MO25a/STRADa	001,2	CAR_11CBS-0187	25	6,7	0,3	RB-CTF	30	2,0

** Maximal molar enzyme assay concentrations, implying enzyme preparations exclusively containing 100 % active enzyme.*

Protein Kinase Assay

A radiometric protein kinase assay (33PanQinase® Activity Assay) was used for measuring the kinase activity of the nine protein kinases. All kinase assays were performed in 96-well ScintiPlatesTM from PerkinElmer (Boston, MA, USA) in a 50 µl reaction volume. The reaction cocktail was pipetted in 4 steps in the following order:

- 25 µl of assay buffer (standard buffer/[γ-33P]-ATP)
- 10 µl of ATP solution (in H2O)
- 5 µl of test compound (in 10 % DMSO)
- 10 µl of enzyme/substrate mixture

The reaction cocktails were incubated at 30ºC for 60 minutes. The reaction was stopped with 50 µl of 2 % (v/v) H_3PO_4, plates were aspirated and washed two times with 200 µl 0.9 % (w/v) NaCl. Incorporation of 33Pi was determined with a microplate scintillation counter (Microbeta, Wallac).

Evaluation of Raw Data

The median value of the counts in column 1 (n = 8) of each assay plate was defined as "low control". This value reflects unspecific binding of radioactivity to the plate in the absence of a protein kinase but in the presence of the substrate. The median value of the counts in column 2 of each assay plate (n = 8) was taken as the "high control", i.e. full activity in the absence of any inhibitor. The difference between high and low control was taken as 100 % activity.

As part of the data evaluation the low control value from a particular plate was subtracted from the high control value as well as from all 80 "compound values" of the corresponding plate. The residual activity (in %) for each well of a particular plate was calculated by using the following formula:

Res. Activity (%) = 100 X [(cpm of compound – low control) / (high control – low control)]

2.3. Comparative analysis of the binding between compounds 2 and 12

A comparative analysis of the docking binding modes of compounds **2** [3] and **12** was performed to investigate how the main structural difference between these two compounds—namely, the linker connecting the purine and benzoxazine rings—affects their binding to hHER2. In **2**, this linker consists of an ethylene chain, whereas in **12** it is reduced to a methylene group.

For (*S*)-**2** docked into hHER2 (PDB ID: 3RCD), the purine moiety is positioned within the hinge region, forming a hydrogen bond with the backbone of Met801. The benzoxazine ring projects toward the solvent-exposed area and the pocket at the entrance of the catalytic site, establishing a hydrogen bond with Cys805. The phenylsulfonamide moiety, however, is oriented toward the catalytic cavity rather than exposed to solvent. In contrast, in (*S*)-**12** the purine is rotated outward and oriented toward the solvent-exposed region, adopting a U-shaped conformation with the phenylsulfonamide moiety. The benzoxazine ring occupies the hinge region, flanked by hydrophobic residues Leu726, Val734, Leu800, Met801, and Leu852, while the phenylsulfonamide is directed toward the catalytic site, though its exposure to solvent is partially shielded by the pyrrolopyrimidine (Figure S3a).

In (*R*)-**12**, the pyrrolopyrimidine ring is displaced toward the solvent-exposed area, where it forms a hydrogen bond with Cys805. The benzoxazine ring, instead, is accommodated within the hinge region, and the phenylsulfonamide moiety extends into a hydrophobic pocket inside the catalytic site formed by Thr798 and the aliphatic side chain of Lys753. In contrast, (*R*)-**2** adopts an extended conformation in which the pyrrolopyrimidine ring is inserted into the hinge region, forming a hydrogen bond with Met801, while the benzoxazine ring folds into the catalytic cavity in close contact with the pyrrolopyrimidine, generating an internal U-shaped arrangement. In this configuration, the phenylsulfonamide moiety projects outward toward the solvent-exposed region (Figure S3b).

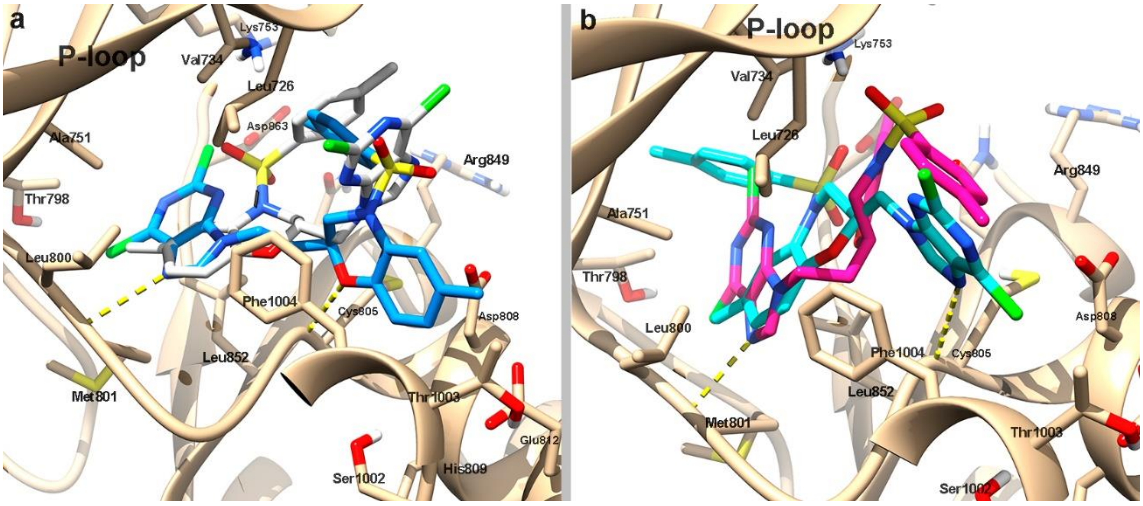

Figure S3. Predicted binding poses of (**a**) (*S*)-**2** (blue) and (*S*)-**12** (white); (**b**) (*R*)-**2** (pink) and (*R*)-**12** (cyan), on hHER2 (pdb ID 3RCD, tan). Hydrogen bonds are represented as dashed yellow lines.

A similar analysis was conducted for hJNK1 (PDB ID: 4AWI). For the (S)-enantiomers, both ligands place the pyrrolopyrimidine ring within the catalytic cavity, oriented toward a pocket bounded by Val40, Met108, and Lys55 on one side and by Gln37 and Leu168 on the opposite side. In (*S*)-**2**, the pyrrolopyrimidine additionally engages Lys55 through a hydrogen bond. The main difference lies in the relative orientations of the benzoxazine and phenylsulfonamide groups. In (*S*)-**2**, the benzoxazine occupies the hinge region and the phenylsulfonamide projects toward the solvent-exposed area. In (*S*)-**12**, by contrast, the arrangement is reversed; the benzoxazine is positioned at the outer face of the hinge and forms a hydrogen bond with Asn114 (Figure S4a).

For the (*R*)-enantiomers, the docked conformations of the two compounds are largely superimposable, especially for the benzoxazine and phenylsulfonamide fragments. The benzoxazine ring and the sulfonyl group occupy the deeper part of the hinge region, whereas the phenyl ring of the phenylsulfonamide points toward the solvent-exposed exterior. In both cases, a hydrogen bond is observed between the protein backbone of Met111 and one of the sulfone oxygens. The principal difference resides in the orientation of the pyrrolopyrimidine ring: in both (*R*)-2 and (*R*)-12 it sits in the inner portion of the ATP pocket, yet in (*R*)-2 it adopts an orientation roughly perpendicular to that in (*R*)-12, plausibly reflecting the reduced reach imposed by the shorter methylene linker (Figure S4b).

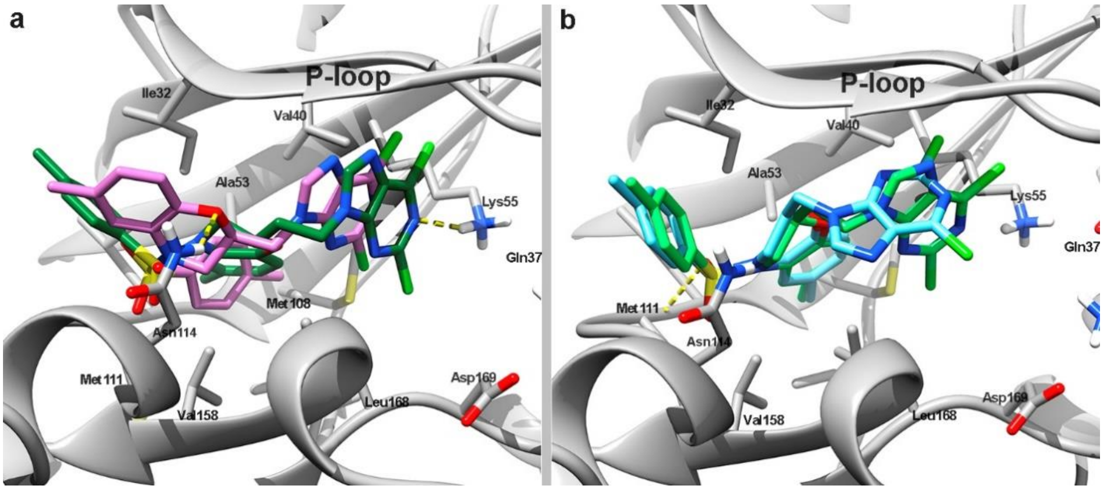

Figure S4. Predicted binding poses of (**a**) (*S*)-**2** (green) and (*S*)-**12** (wine); (**b**) (*R*)-**2** (light green) and (*R*)-**12** (cyan), on hJNK1 (pdb ID 4AWI, grey). Hydrogen bonds are represented as dashed yellow lines.

Taken together, these docking results suggest that the binding modes of **2** and **12** are shaped by both linker length and the structural context of the kinase ATP pocket. In hHER2, the hinge residue Met801 and the surrounding gatekeeper Thr798 create a relatively open environment [4,5,6]. Under these conditions, the ethylene linker of **2** enables the pyrrolopyrimidine to engage the hinge, while the benzoxazine and phenylsulfonamide are redirected toward the catalytic cavity or solvent-facing entrance. By contrast, the shorter methylene linker in **12** restricts conformational flexibility, resulting in hinge engagement by the benzoxazine and displacement of the pyrrolopyrimidine toward solvent.

In hJNK1, the hinge residues Met111 and Asn114 define a more compact adenine-binding environment, with additional contributions from Lys55, Val40, Met108, and Leu168 [7,8]. Both compounds can achieve hinge interactions, but the reduced linker length in **12** constrains the scaffold, producing an alternative pose in which the pyrrolopyrimidine ring is oriented nearly perpendicular to that in **2**. These differences are consistent with structural studies reporting that hinge-binding motifs in JNK1 are sensitive to small variations in substituent geometry and linker reach [7,8].

3. References

[1] Singh, S. N.; Jayaprakash, S.; Venkateshwara Reddy, K.; Nakhi, A.; Pal, M. A metal catalyst-free and one-pot synthesis of (3,4-dihydro-2*H*-benzo[b][1,4]oxazin-2-yl)methanol derivatives in water *RSC Adv.* **2015**, 5, 84889-84893. DOI: 10.1039/c5ra14478g

[2] Bourlot, A.-S.; Guillaumet, G.; Mérour, J.-Y. A straightforward route to 4*H*-1,4-Benzoxazine-2-carbaldehydes by swern oxidation. *J. Heterocycl. Chem.* **1996**, *33*, 191-196. DOI:10.1002/jhet.5570330133

[3] Conejo-García, A.; Jiménez-Martínez, Y.; Cámara, R.; Franco-Montalbán, F.; Peña-Martín, J.; Boulaiz, H.; Carrión, M. D. New substituted benzoxazine derivatives as potent inducers of membrane permeability and cell death. *Bioorg. Med. Chem.* **2024**, 111, 117849. DOI: 10.1016/j.bmc.2024.117849

[4] Aertgeerts, K.; Skene, R.; Yano, J.; Sang, B.-C.; Zou, H.; Snell, G.; Jennings, A.; Iwamoto, K.; Habuka, N.; Hirokawa, A.; *et al.* Structural Analysis of the Mechanism of Inhibition and Allosteric Activation of the Kinase Domain of HER2 Protein. *J. Biol. Chem***. 2011,** *286*, 18756-18765. DOI: 10.1074/jbc.M110.206193.

[5] Collins, D. M.; Conlon, N. T.; Kannan, S.; Verma, C. S.; Eli, L. D.; Lalani, A. S.; Crown, J. Preclinical Characteristics of the Irreversible Pan-HER Kinase Inhibitor Neratinib Compared with Lapatinib: Implications for the Treatment of HER2-Positive and HER2-Mutated Breast Cancer. *Cancers*, **2019,** 11, 737. DOI: 10.3390/cancers11060737.

[6] Son, J.; Jang, J.; Beyett, T. S.; Eum, Y.; Haikala, H. M.; Verano, A.; *et al.* A Novel HER2-Selective Kinase Inhibitor Is Effective in HER2 Mutant and Amplified Non–Small Cell Lung Cancer. *Cancer Res.* **2022**, *82*, 1633-1645. DOI: 10.1158/0008-5472.CAN-21-2693.

[7] Heo, Y.-S.; Kim, S. K.; Seo, C. I.; Kim, Y. K.; Sung, B.-J.; Lee, H. S.; Lee, J. I.; Park, S.-Y.; Kim, J. H.; Hwang, K. Y.; *et al.* Structural Basis for the Selective Inhibition of JNK1 by the Scaffolding Protein JIP1 and SP600125. *EMBO J.*, **2004,** *23*, 2185–2195. DOI: 10.1038/sj.emboj.7600212.

[8] Yang, R.; Zhao, G.; Yan, B. Discovery of Novel c-Jun N-Terminal Kinase 1 Inhibitors from Natural Products: Integrating Artificial Intelligence with Structure-Based Virtual Screening and Biological Evaluation. *Molecules* **2022,** *27*, 6249. DOI: 10.3390/molecules27196249.
